# Spatial proteomics reveals signal sequence characteristics correlated with localization in cyanobacteria

**DOI:** 10.1093/plphys/kiaf186

**Published:** 2025-08-06

**Authors:** Kelsey Dahlgren, Christopher C Ebmeier, Emily Koke, Jeffrey C Cameron

**Affiliations:** Department of Biochemistry, University of Colorado, Boulder, CO 80309, USA; Renewable and Sustainable Energy Institute, University of Colorado, Boulder, CO 80303, USA; BioFrontiers Institute, University of Colorado, Boulder, CO 80303, USA; Interdisciplinary Quantitative Biology Program, BioFrontiers Institute, University of Colorado, Boulder, CO 80303, USA; Department of Biomedical Sciences and Pathobiology, Virginia Tech, Blacksburg, VA 24061, USA; Proteomics and Mass Spectrometry Core Facility, Department of Biochemistry, University of Colorado, Boulder, CO 80303, USA; Department of Biochemistry, University of Colorado, Boulder, CO 80309, USA; Department of Biochemistry, University of Colorado, Boulder, CO 80309, USA; Renewable and Sustainable Energy Institute, University of Colorado, Boulder, CO 80303, USA; National Renewable Energy Laboratory, Golden, CO 80401, USA

## Abstract

Cyanobacteria have an inner and outer cell membrane enclosing the periplasm and cell wall and an additional set of internal membranes (called the thylakoid membranes) enclosing the thylakoid lumen. The periplasm and thylakoid lumen have unique proteomes, but the mechanisms regulating protein sorting to these locations have remained elusive. Here, proximity-based proteomics using the engineered peroxidase APEX2 was performed in the cyanobacteria *Synechococcus* sp. PCC 7002 to profile the proteomes of the cytoplasm, thylakoid lumen, and the periplasm and outer membrane (P-OM). Our analyses revealed specific roles for the thylakoid lumen in photosynthesis and energy generation, as well as roles for the periplasm in metabolite transport and binding, cell motility, and cell wall maintenance. Forty proteins localized to both the thylakoid lumen and the P-OM; however, their biological functions remain unclear. We also analyzed the correlation between signal sequence characteristics and differential protein localization to either the thylakoid lumen or the P-OM. In PCC 7002, as well as *Synechocystis* sp. PCC 6803 and *Nostoc* sp. PCC 7120, thylakoid lumen proteins translocated across membranes via the Secretory (Sec) system possessed more hydrophobic and alpha-helical signal sequence H-regions than P-OM proteins. The signal sequences of homologous proteins in *Gloeobacter violaceus* PCC 7421, a cyanobacterial species with a combined thylakoid lumen and periplasmic space, did not exhibit such differences. Therefore, the pattern of increased H-region hydrophobicity and alpha helix content is specific to cyanobacteria with a separate thylakoid lumen space and likely contributes to proper protein sorting between the thylakoid lumen and periplasm.

## Teaser

Proximity-based proteomics reveal sequence features associated with protein localization to distinct membrane-bound compartments in cyanobacteria, including the periplasm and thylakoid lumen.

## Introduction

**Figure 1.**
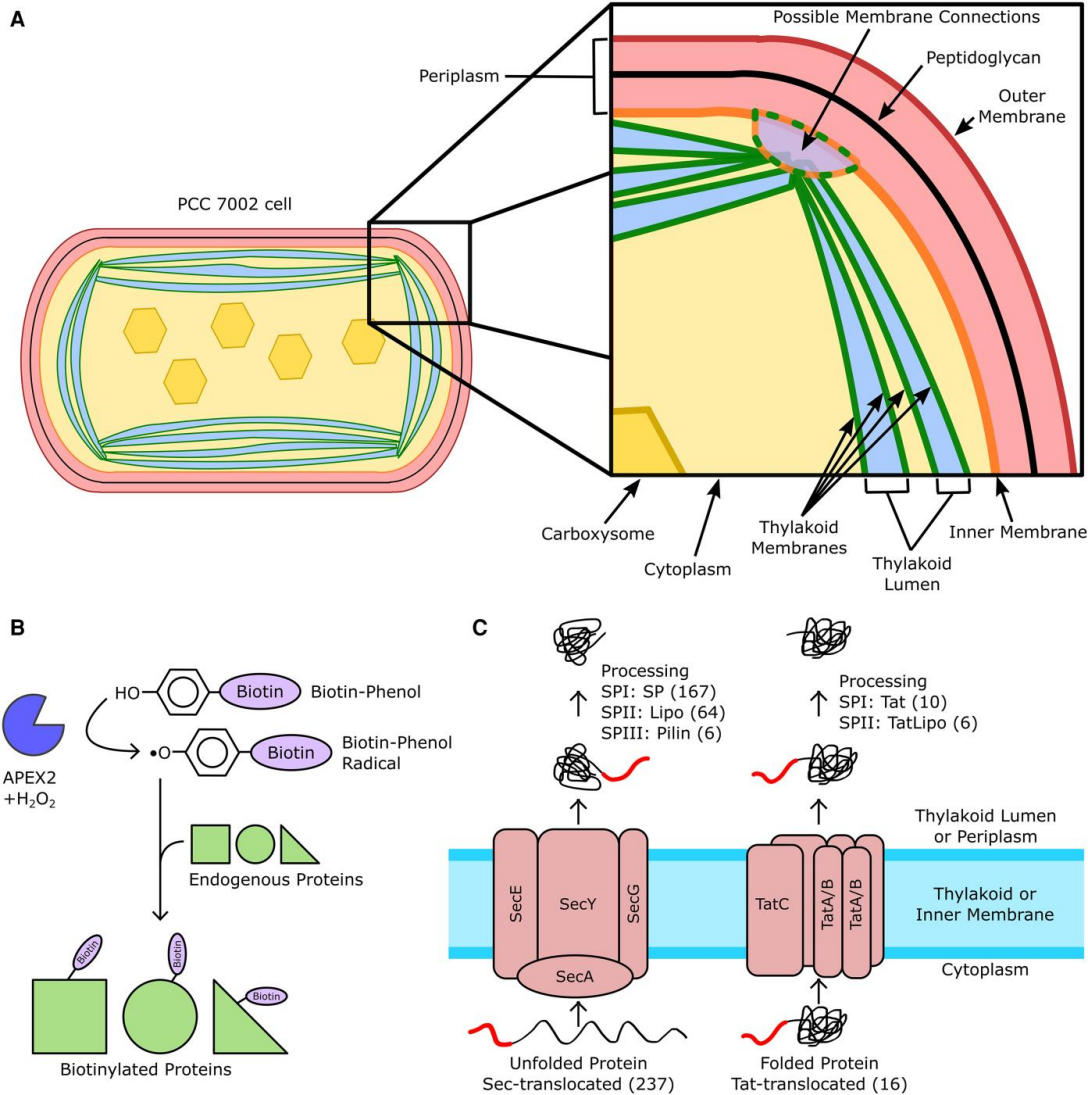
PCC 7002 cellular structure, protein translocation across membranes, and APEX2 labeling. **A)** A diagram of PCC 7002 cellular structure with an inset showing greater detail of the membrane structure. The hexagons in the cytoplasm are carboxysomes. The periplasmic space contains the cell wall and is bordered by the inner and outer cell membranes. The TMs enclose the thylakoid lumen. There may be transient membrane connections between the inner and TMs, which are shown in the inset. **B)** In the presence of BP and H_2_O_2_, APEX2 catalyzes a reaction creating a BP radical. The BP radical covalently labels endogenous proteins. **C)** The Sec pathway translocates unfolded proteins and the Tat pathway translocates folded proteins. The N-terminal signal peptide (shown as a thicker red line) is cleaved from the processed protein with a Type I, II, or III signal peptidase (SPI, SPII, and SPIII, respectively). The numbers in parenthesis represent the number of proteins in PCC 7002 predicted to utilize the translocation pathway (below membrane) or the signal peptide cleavage type in combination with the translocation pathway (above membrane) by SignalP 6.0.

Cyanobacteria are oxygenic phototrophs that play a crucial role in the carbon cycle and other globally important biogeochemical processes through production of O_2_ and fixation of N_2_. Like other Gram-negative bacteria, cyanobacteria have a plasma membrane (PM; also referred to as “inner membrane”) and outer membrane (OM) that enclose a periplasmic space containing the peptidoglycan cell wall. Most extant cyanobacteria also contain thylakoid membranes (TMs), a set of intracellular membranes that house the photosynthetic machinery and enclose the thylakoid lumen, the site of water oxidation by light-dependent water-quinone oxidoreductase, PSII (Mareš et al. 2019). However, several strains of cyanobacteria lacking distinct TMs have been isolated (Rexroth et al. 2011; Li et al. 2020). In these strains, the photosynthetic machinery is localized in the PM, and the periplasm functions as the thylakoid lumen. A PM origin for the internalized TM has been postulated based on the identification of early PSII assembly intermediates in the inner membrane (Zak et al. 2001). However, the mechanism of TM biogenesis is still poorly understood and widely debated (Mechela et al. 2019). All free-living cyanobacteria also contain carboxysomes, protein-based organelles that function as key components of their highly efficient CO_2_-concentrating mechanism (Cameron et al. 2013; Rae et al. 2013) (Fig. 1A).

Given the complex cell biology of cyanobacterial cells and many outstanding questions regarding the biogenesis of the TMs, numerous studies have employed traditional biochemical fractionation techniques to elucidate the proteomes of the inner membrane, OM, and TM in the model cyanobacterial species *Synechocystis* sp. PCC 6803 (PCC 6803) (Wang et al. 2000; Huang et al. 2002; Herranen et al. 2004; Huang et al. 2004; Srivastava et al. 2005; Huang et al. 2006; Pisareva et al. 2007; Rajalahti et al. 2007; Zhang et al. 2009; Agarwal et al. 2010; Rowland et al. 2010; Pisareva et al. 2011; Li et al. 2012; Liberton et al. 2016; Baers et al. 2019; Wang et al. 2022). Although multiple studies have determined the proteins in the periplasmic space (Fulda et al. 2000; Kurian et al. 2006b; Rajalahti et al. 2007; Wang et al. 2022), the proteomes of other membrane-enclosed spaces in cyanobacteria have remained elusive. Recently, we described the proteome of the thylakoid lumen (Dahlgren et al. 2021). To accomplish this, we performed proximity-based proteomics in the model cyanobacterium *Synechococcus* sp. PCC 7002 (PCC 7002) by expressing an engineered ascorbate peroxidase (APEX2) fused to PsbU, a known extrinsic PSII protein that is localized to the thylakoid lumen (Shen et al. 1997; Nishiyama et al. 1999). Using biotin-phenol (BP) and H_2_O_2_ as substrates, APEX2 catalyzes a reaction producing a highly reactive biotin-phenol radical, which covalently binds to proteins within a 10- to 20-nm radius from the site of production (Fig. 1B) (Rhee et al. 2013; Lam et al. 2015; Hung et al. 2016). The biotin-phenol radical does not cross the membrane, allowing for specific labeling of membrane-bound proteomes (Rhee et al. 2013). Furthermore, APEX2 functions in multiple cellular compartments (Lam et al. 2015; Hung et al. 2016 ), and recent studies have demonstrated the ability to identify PSII-proximal proteins in PCC 6803 (Xiao et al. 2022) using a similar approach to ours (Dahlgren et al. 2021 ). In contrast to biochemical fractionation techniques that require cell lysis prior to enrichment of proteins, APEX2-based proteomics enables rapid (<1 min) in vivo labeling of proteins in their native compartments before cell lysis, minimizing contamination and ambiguous cellular localization.

The multiple membrane-enclosed compartments in cyanobacteria necessitate transport and sorting mechanisms to ensure that proteins are targeted to the correct cellular location. N-terminal signal sequences are known to be utilized to export soluble proteins to both the periplasm and the thylakoid lumen (Mackle and Zilinskas 1994). Therefore, signal sequences contain the information needed to target proteins to the thylakoid lumen or periplasm in cyanobacteria. Signal sequences interact with translocation machinery to facilitate translocation to the proper region before cleavage from the processed protein by a signal peptidase (Frain et al. 2016). Type I, Type II, and Type III signal peptidases (SPI, SPII, and SPIII, respectively), which each cleave different sequence motifs, are present in cyanobacteria. Cyanobacteria possess 2 systems to translocate soluble proteins containing signal peptides across membranes, the secretory (Sec) pathway to translocate unfolded proteins and the twin arginine translocation (Tat) pathway to translocate folded proteins (Fig. 1C) (Frain et al. 2016). However, most cyanobacteria possess only one set of genes for each of the Sec and Tat translocation systems, and they are both localized to the PM and TM (Russo and Zedler 2020), raising questions about how proteins are specifically targeted to the correct compartment. We hypothesized that signal sequences contain characteristics that can be differentiated directly or indirectly by translocation complexes present in the PM and TM and sought to identify these features in order to better understand protein targeting and its contributions to the subcellular organization of cyanobacteria. To accomplish this, we performed a comprehensive and quantitative analysis of the “compartmentalome,” defined here as the proteome of each membrane-bound compartment, in PCC 7002. These data are available in the form of a searchable website to the community at https://dahlgren-lab.github.io/cyano-compartmentalome-data/. We used the compartmentalome dataset generated in this paper to identify signal sequence characteristics correlated with localization in the thylakoid lumen or the periplasm. Using the thylakoid lumen and periplasm proteomes in PCC 7002, we identified protein homologs in other species of cyanobacteria and analyzed their signal sequences for evolutionarily conserved characteristics associated with localization. Furthermore, we identified a subset of proteins present in both the periplasm and thylakoid lumen, providing further support for the model that the TM is derived from the PM in cyanobacteria. Together, these results give rise to a model for protein targeting in cyanobacteria and insights into the TM biogenesis pathway.

## Results

### Targeting APEX2 to different membrane-bound compartments

**Figure 2.**
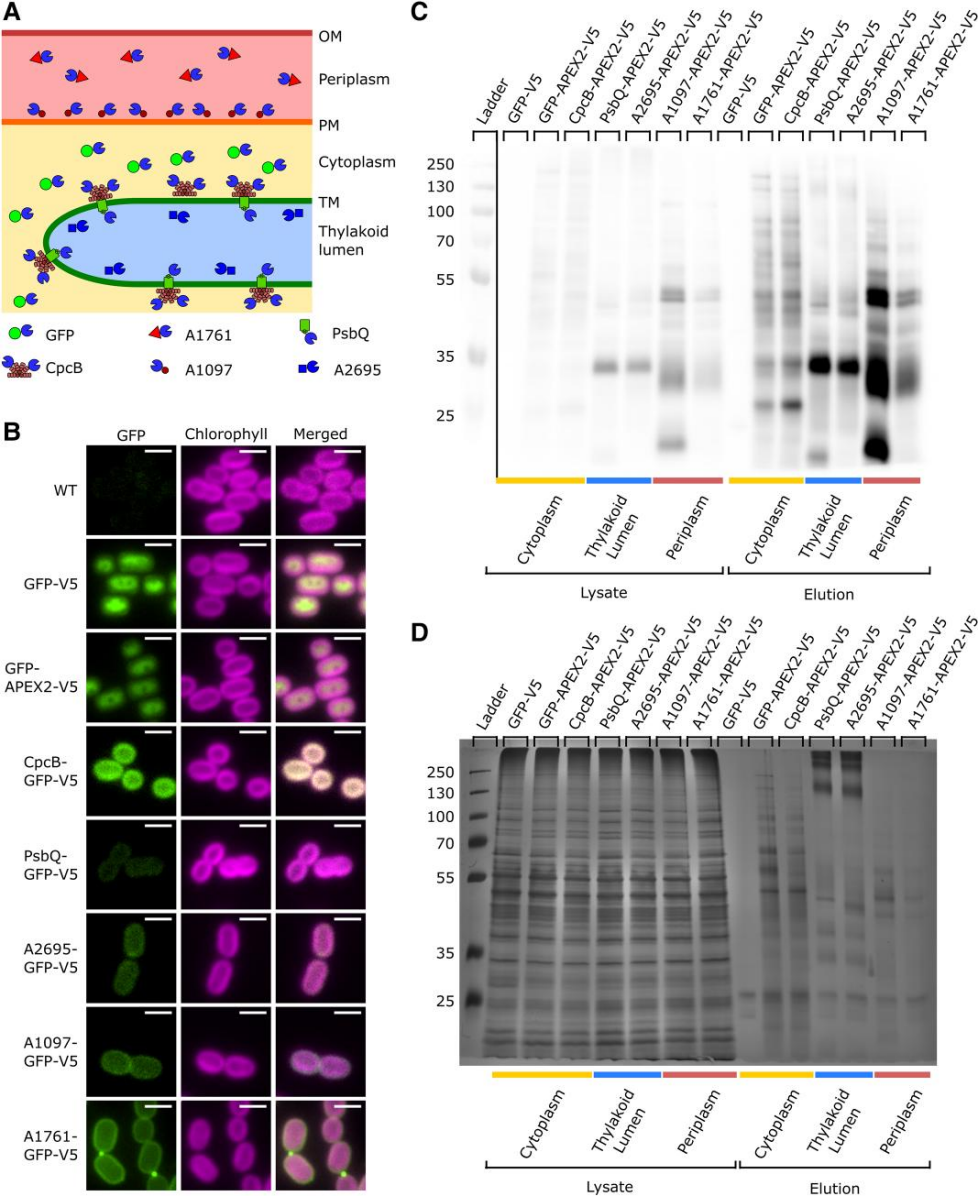
Localization and labeling activity of APEX2 fusion proteins in strains used for proteomics. **A)** Diagram of the localization of APEX2 fusion proteins used to identify the proteome of the cytoplasm, thylakoid lumen, and periplasm. CpcB, PsbQ, and A1097 fusion proteins are membrane associated in the cytoplasm, thylakoid lumen, and periplasm, respectively. In contrast, GFP, A2695, and A1761 are soluble proteins in the cytoplasm, thylakoid lumen, and periplasm, respectively. **B)** Localization of GFP fusion proteins analogous to the APEX2 fusion proteins used to label proteomes for MS. GFP fluorescence is in the left column, chlorophyll fluorescence is in the middle column, and a merged image with both GFP and chlorophyll fluorescences is shown in the right column. The strain label is to the left of the images. All images have normalized lookup tables for the chlorophyll channel. Wild type (WT) PCC 7002, CpcB-GFP-V5, PsbQ-GFP-V5, A2695-GFP-V5, and A1097-GFP-V5 have a normalized lookup table for GFP. GFP-V5, GFP-APEX2-V5, and A1761-GFP-V5 lookup tables were set with a higher maximum to better visualize protein localization. Scale bars are 2 *µ*m. Biotinylation patterns of APEX2 localized in different membrane-bound compartments are shown on an **C)** antibiotin blot or **D)** silver stain. The ladder with molecular weights in kDa is the far left lane. The next 7 lanes show biotinylation patterns present in cell lysate after APEX2-dependent biotinylation. The 7 lanes on the right are biotinylation patterns of purified biotinylated proteins from the cell lysate eluted from streptavidin beads. The cellular localization of the protein constructs is labeled on the bottom of the gel.

APEX2 was targeted to the cytoplasm, thylakoid lumen, and periplasm as a C-terminal tag on bait proteins to produce strains for proximity-based proteomics in PCC 7002. The APEX2 fusion proteins were integrated into a neutral site in the genome and expressed using a constitutive promoter. We tested ∼50 different bait proteins to identify candidates for proteomics using stringent criteria based on (i) localization data, (ii) optimal APEX2 activity, and (iii) experimental reproducibility (Supplementary Figs. S1 and S2 and Text). To ensure a robust and representative proteome of each membrane-bound region, we chose a membrane-associated (CpcB, PsbQ, and A1097) and a soluble (GFP, A2695, and A1716) protein that met our selection criteria as bait (Fig. 2A).

A schematic representation of APEX2 fusion proteins used for proteomics in this study is featured in Fig. 2. GFP-APEX2 is soluble in the cytoplasm. CpcB, a rod subunit of the phycobilisomes, an antenna complex involved in light absorption that complexes with and transfers light energy to PSI and PSII, is closely associated with the cytoplasmic side of the TMs (Chang et al. 2015; Zheng et al. 2021). Both PsbQ and A2695 were identified in the thylakoid lumen proteome of PCC 7002 (Dahlgren et al. 2021 ) and were used to target APEX2 to the thylakoid lumen. PsbQ is an extrinsic lumenal subunit of PSII with a lipoprotein modification (Kashino et al. 2002, 2006; Roose et al. 2007 ), and A2695 is a soluble thioredoxin homologous to Slr1796 in PCC 6803 (Zhu et al. 2016). A1097, sometimes referred to as PetC3, is a Rieske FeS lipoprotein involved in cell envelope homeostasis used to target APEX2 to the periplasmic side of the inner membrane (Veit et al. 2016). A1761, annotated as a periplasmic Zn-dependent hydrolase, targeted APEX2 to the soluble fraction of the periplasm (McClure et al. 2016).

Several techniques were used to verify protein localization to a specific membrane-bound compartment. First, we used fluorescence microscopy to verify localization using C-terminal GFP fusions (Fig. 2B). GFP-APEX2 localized to the cytoplasm, and CpcB-GFP was associated with the TMs on the cytoplasmic side. GFP fusions of PsbQ and A2695 were associated with chlorophyll fluorescence representing the TMs and thylakoid lumen. A ring of GFP signal around the chlorophyll fluorescence localized fusions of A1097 and A1761 to the periplasm. Next, immunofluorescence microscopy was performed against the V5 epitope on APEX2 (Supplementary Fig. S1). The localization results were similar to those obtained from GFP fusions of each protein, confirming expected localizations. Finally, the activity of each APEX2 was tested by performing APEX2-dependent biotinylation and visualizing the pattern of biotinylated proteins separated on an SDS-PAGE gel (Fig. 2, C and D; Supplementary Fig. S2).

### APEX2-dependent biotinylation for proteomics

APEX2-dependent biotinylation was performed using 6 cell lines expressing APEX2 fusion proteins (GFP, CpcB, PsbQ, A2695, A1097, and A1761) along with a cell line expressing just GFP as a negative control. Each cell line expressing an APEX2 fusion protein efficiently biotinylated endogenous proteins (Fig. 2, C and D). The biotinylated proteins were then purified on streptavidin beads (Fig. 2, C and D). To determine the proteome of the cytoplasm, thylakoid lumen, and periplasm, APEX2-dependent labeling was performed in 3 replicate cultures of each APEX2 fusion protein. Each replicate was lysed, and biotinylated proteins were purified on streptavidin beads. Immunoblots of the lysates from each replicate and silver stains of the biotinylated elution from each replicate are shown in Supplementary Fig. S3.

### Mass Spectrometry of biotinylated proteins and quantitative proteomics

To prepare each replicate for mass spectrometry (MS), proteins bound to streptavidin beads were eluted, reduced, alkylated, and digested to produce peptides. Peptides from each replicate were then labeled with a unique Tandem Mass Tag (TMT) from the TMTpro 18-plex kit to allow for quantitative proteomics (Li et al. 2021). All 18 samples were combined (3 replicates from each of the 6 APEX2 protein fusions), separated on a column, and run on an Orbitrap (Thermo Fisher Scientific) mass spectrometer to identify peptides. The ratios of TMT signals were used to quantify the relative amount of every unique peptide from each sample. These values were normalized between samples and used to quantify the relative amount of each protein present in each replicate of each APEX2 fusion protein. A total of 1687 proteins were identified out of the 3179 proteins in the UniProt reference proteome of PCC 7002. The 1,687 proteins represent 53% of all proteins predicted to be encoded in the genome of PCC 7002, a coverage comparable with Wang et al. (2022) (55%) and Baers et al. (2019) (66.5%), existing large-scale proteomics studies in PCC 6803.

### Increased correlation between samples in similar localizations

**Figure 3.**
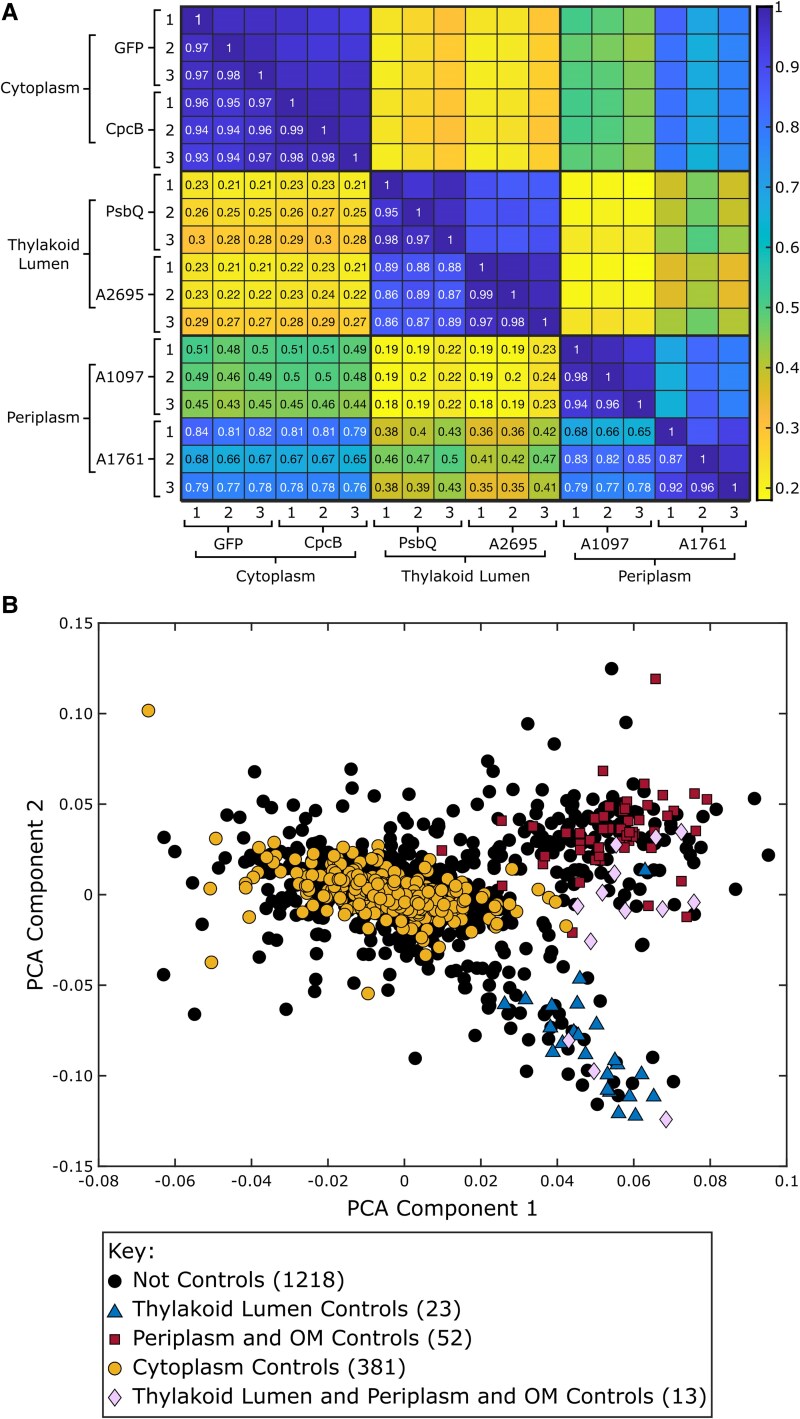
Proteins within the same membrane-bound compartment cluster together. **A)** A heatmap displays Pearson correlation coefficients calculated between samples submitted to MS using normalized MS values. The key for the heatmap is on the right of the table. The bottom left half of the heatmap shows the correlation coefficients. The replicate number, protein fused to APEX2 for targeting, and the localization of the APEX2 fusion proteins are listed on the bottom and left of the heatmap. **B)** A principal component analysis (PCA) plot displays clustering between control proteins with known cellular localizations. Log-transformed normalized MS values for all 1,687 proteins identified by MS were used to make the PCA plot. Different groups of control proteins are shown in different colors and shapes, with the key below the PCA plot.

The Pearson's correlation coefficient was calculated using the TMT reporter ion intensity values for each protein to determine similarity between samples (Fig. 3A). Replicates of the same APEX2 fusion proteins exhibited high correlation coefficients (>0.87). Similarly, the correlations between replicates localized in the same membrane-bound compartment were also high. For example, cytoplasmic CpcB and GFP-APEX2 fusion proteins (>0.92) and lumenal PsbQ and A2695-APEX2 fusion proteins (>0.86) exhibited high coefficients. The periplasmic A1097 and A1761-APEX2 fusion proteins exhibited lower coefficients when compared with each other (>0.65). As expected, correlation within specific membrane-bound compartments was high, while correlation between membrane-bound compartments was lower as they contained distinct proteomes. A principal component analysis (PCA) plot of the samples also showed clustering between samples localized to the same membrane-bound compartment (Supplementary Fig. S4).

### Building a database of PCC 6803 protein localizations to identify proteins with specific localizations

To analyze the MS data with the method used by Hung et al. (2016) and Dahlgren et al. (2021), proteins with known localizations were used as positive controls to determine enrichment cutoff values necessary for robust categorization of unknown proteins. To accomplish this, a database (Supplementary Table S1) of known protein localizations in cyanobacteria was needed. The database included proteomics studies, as well as studies of individual proteins, the majority of which are from PCC 6803 (Zak et al. 1999, 2001; Choi et al. 2000, 2020; Fulda et al. 2000, 2002, 2006; Sergeyenko and Los 2000; Wang et al. 2000, 2009, 2022; Huang et al. 2002, 2004, 2006; Kashino et al. 2002, 2006; Ohkawa et al. 2002; Simon et al. 2002; Lindahl and Florencio 2003; Herranen et al. 2004; Klinkert et al. 2004; Zhang et al. 2004, 2009, 2013; Gan et al. 2005; Srivastava et al. 2005; Komenda et al. 2006; Kurian et al. 2006b, 2006a; Pérez-Pérez et al. 2006; Slabas et al. 2006; Mata-Cabana et al. 2007; Pisareva et al. 2007, 2011; Rajalahti et al. 2007; Aldridge et al. 2008; Xu et al. 2008; Boehm et al. 2009; Gao et al. 2009, 2014a, 2014b, 2015; Pandhal et al. 2009; Schultze et al. 2009; Agarwal et al. 2010; Kwon et al. 2010; Rowland et al. 2010, 2011; Zanetti et al. 2010; Rengstl et al. 2011; Wegener et al. 2011; Li et al. 2012; Roberts et al. 2012; Fuszard et al. 2013; Trautner and Vermaas 2013; Mehta et al. 2014; Mikkat et al. 2014; Cheregi et al. 2015; Plohnke et al. 2015; Sacharz et al. 2015; Dong et al. 2016; Heinz et al. 2016; Liberton et al. 2016; Oliveira et al. 2016; Selão et al. 2016; Zhu et al. 2016; Baers et al. 2019; Xiao et al. 2022).

Additional details include the number of studies where a protein was localized to a specific region of interest, the number and position of transmembrane helices, and predicted signal sequences for each PCC 6803 protein, as well as the best reciprocal BLAST hits (orthologs) between PCC 6803 and PCC 7002 proteins. This dataset is available at https://dahlgren-lab.github.io/cyano-compartmentalome-data/. The PCC 7002 proteins orthologous to PCC 6803 with known localizations were used as positive controls for each compartment (Supplementary Table S2).

### Quantitative proteomics to determine protein localization

Proteins with known localizations corresponding to the cytoplasm, thylakoid lumen, and periplasm were marked on a PCA plot of log-transformed normalized TMT reporter ion intensity values for each protein (Fig. 3B). Proteins with similar localizations clustered together. Enrichment values for each protein were calculated from log-transformed TMT ratios. The enrichment values of control proteins with known localizations (Hung et al. 2016; Dahlgren et al. 2021) were used to determine whether proteins were localized to the thylakoid lumen, periplasm and outer membrane (P-OM), cytoplasm, TM, and inner membrane in this study (Supplementary Table S3). Together, these represent the entire PCC 7002 membrane-bound compartmentalome.

### Thylakoid lumen proteome analysis

**Figure 4.**
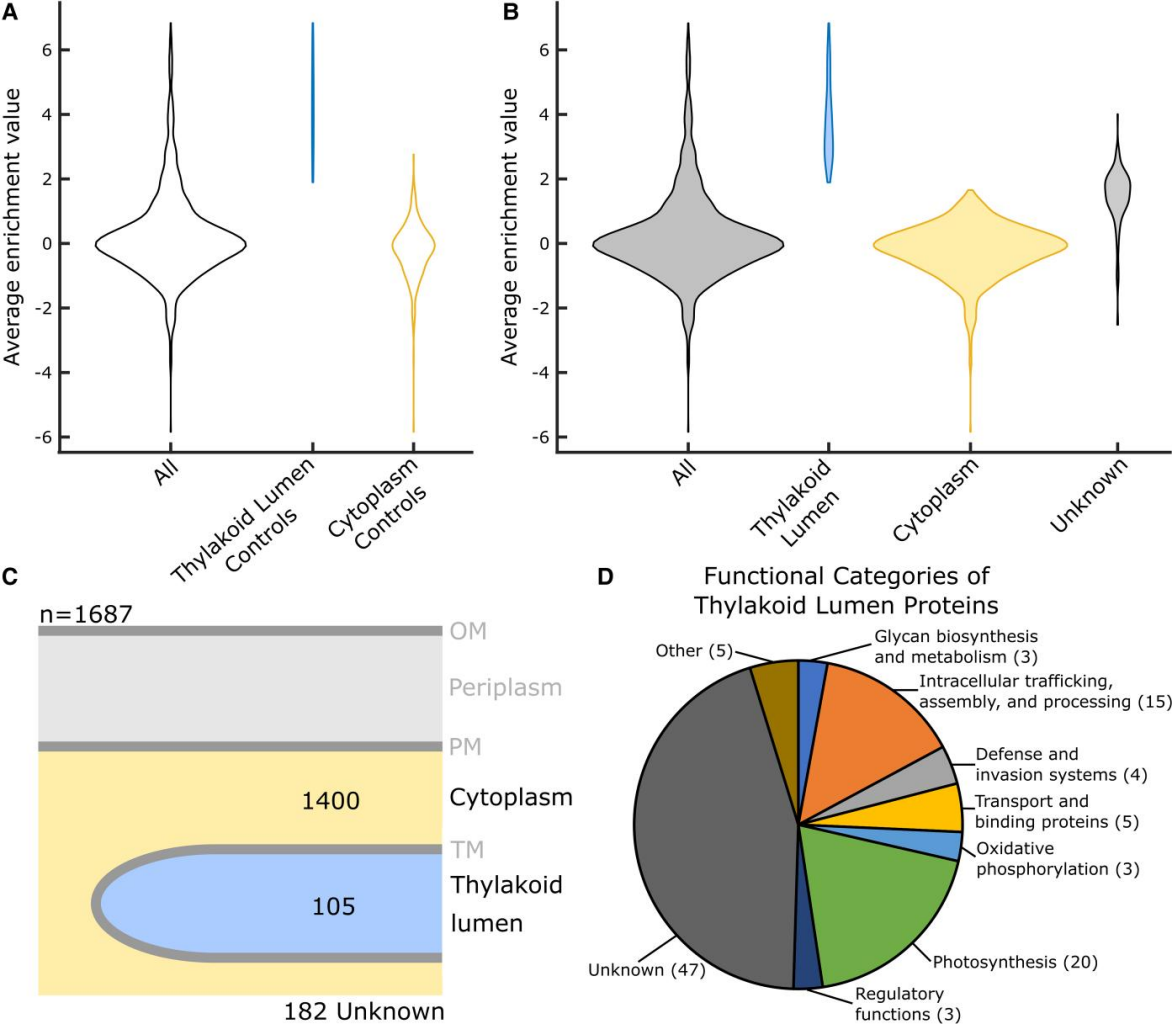
Thylakoid lumen proteome analysis. **A)** Distribution of average enrichment value (log_2_ [lumen/cytoplasm]) for all proteins (*n* = 1,687) and control proteins with known localizations in the thylakoid lumen (*n* = 36) and cytoplasm (*n* = 381). Size of the violins is normalized by the number of samples in each category. **B)** Distribution of average enrichment values (log_2_ [lumen/cytoplasm]) for proteins (*n* = 1,687) localized to the thylakoid lumen (*n* = 105) and cytoplasm (*n* = 1400), as well as proteins that were unable to be confidently localized in this analysis (Unknown) (*n* = 182). Size of the violins is normalized by the number of samples in each category. **C)** An infographic of the protein localizations determined in the analysis of the thylakoid lumen proteome. **D)** The functional categories of proteins localized to the thylakoid lumen proteome in this analysis.

Enrichment values calculated from the TMT reporter ion intensity ratios of thylakoid lumenal APEX2 protein fusions (PsbQ and A2695) and cytoplasmic APEX2 protein fusions (GFP and CpcB) (Supplementary Table S2) revealed substantial differences, allowing us to distinguish the proteomes of these compartments (Fig. 4A). The distribution of mean enrichment values in our dataset is shown in Fig. 4B. One hundred five proteins were localized to the thylakoid lumen, 1,400 proteins were localized to the cytoplasm, and 182 proteins were unable to be confidently localized with this analysis (Fig. 4C). We estimate our coverage of the lumen proteome to be ∼90% based on the identification of 36/40 thylakoid lumen control proteins present in the positive control database.

Seventy-four percent of the thylakoid lumen proteins have previously been localized to the thylakoid lumen or TM in PCC 7002 or PCC 6803 using a variety of methods (Table 1; Supplementary Table S3) (Zak et al. 1999, 2001; Wang et al. 2000, 2022 ; Fulda et al. 2002; Kashino et al. 2002 , 2006 ; Ohkawa et al. 2002; Herranen et al. 2004; Zhang et al. 2004; Srivastava et al. 2005; Komenda et al. 2006 ; Pisareva et al. 2007, 2011; Rajalahti et al. 2007 ; Aldridge et al. 2008 ; Xu et al. 2008; Boehm et al. 2009; Schultze et al. 2009; Agarwal et al. 2010; Rowland et al. 2010 ; Rengstl et al. 2011 ; Wegener et al. 2011 ; Roberts et al. 2012 ; Sacharz et al. 2015; Heinz et al. 2016; Liberton et al. 2016; Selão et al. 2016; Zhu et al. 2016; Baers et al. 2019 ; Dahlgren et al. 2021 ; Xiao et al. 2022 ). Additionally, proteins present in the thylakoid lumen proteome have other characteristics expected for a noncytoplasmic membrane-bound compartment; 88% of proteins in the thylakoid lumen proteome are predicted to have a signal sequence or at least one transmembrane helix (Table 1). The functions of the thylakoid lumen proteins were also analyzed (Fig. 4D). The largest functional category was “proteins with an unknown function” (47, 45%). However, “photosynthesis” (20, 19%), the primary process performed by the thylakoid lumen and TMs, was the second largest functional category of thylakoid lumen proteins (Liberton et al. 2016). PetJ, the soluble electron carrier between cytochrome b_6_f and PSI, and additional proteins from the PSI, PSII, and cytochrome b_6_f protein complexes were identified in our thylakoid lumen proteome. Other thylakoid lumen proteins were involved in oxidative phosphorylation (3, 3%), including subunits of ATP synthase and NADH dehydrogenase. The 3rd largest functional category of thylakoid lumen proteins was “intracellular trafficking, protein assembly, and processing” (15, 14%), which includes both the Sec protein translocation pathway subunit SecE and PSII assembly factors CtpA, Ycf48, PratA, and Psb35. Five “transport and binding proteins” (5%) were also identified in the thylakoid lumen. A comparison of the thylakoid lumen proteome described in this study and the previously described thylakoid lumen proteomes by Dahlgren et al. (2021) and Xiao et al. (2022) is displayed in Supplementary Fig. S5 and discussed in the Supplementary Text.

**Table 1.** Thylakoid lumen proteome information

Category	Number	%
Previous thylakoid lumen localization (homologs included)	74	70.5
In PCC 7002 only	70	66.7
In PCC 6803 (80 total homologs)	48	60^a^
Previous TM or lumen localizations (homologs included)	78	74.3
In PCC 6803 (80 total homologs)	65	81.3^a^
Possess a signal sequence or transmembrane helix	92	87.6
Known thylakoid lumen proteins (36 detected by MS)	36	100^b^

^a^The percentage of proteins with homologs in PCC 6803 (80 total).

^b^The percentage of thylakoid lumen control proteins detected by MS (36 total) in the thylakoid lumen proteome.

### Periplasm and outer membrane proteome analysis

**Figure 5.**
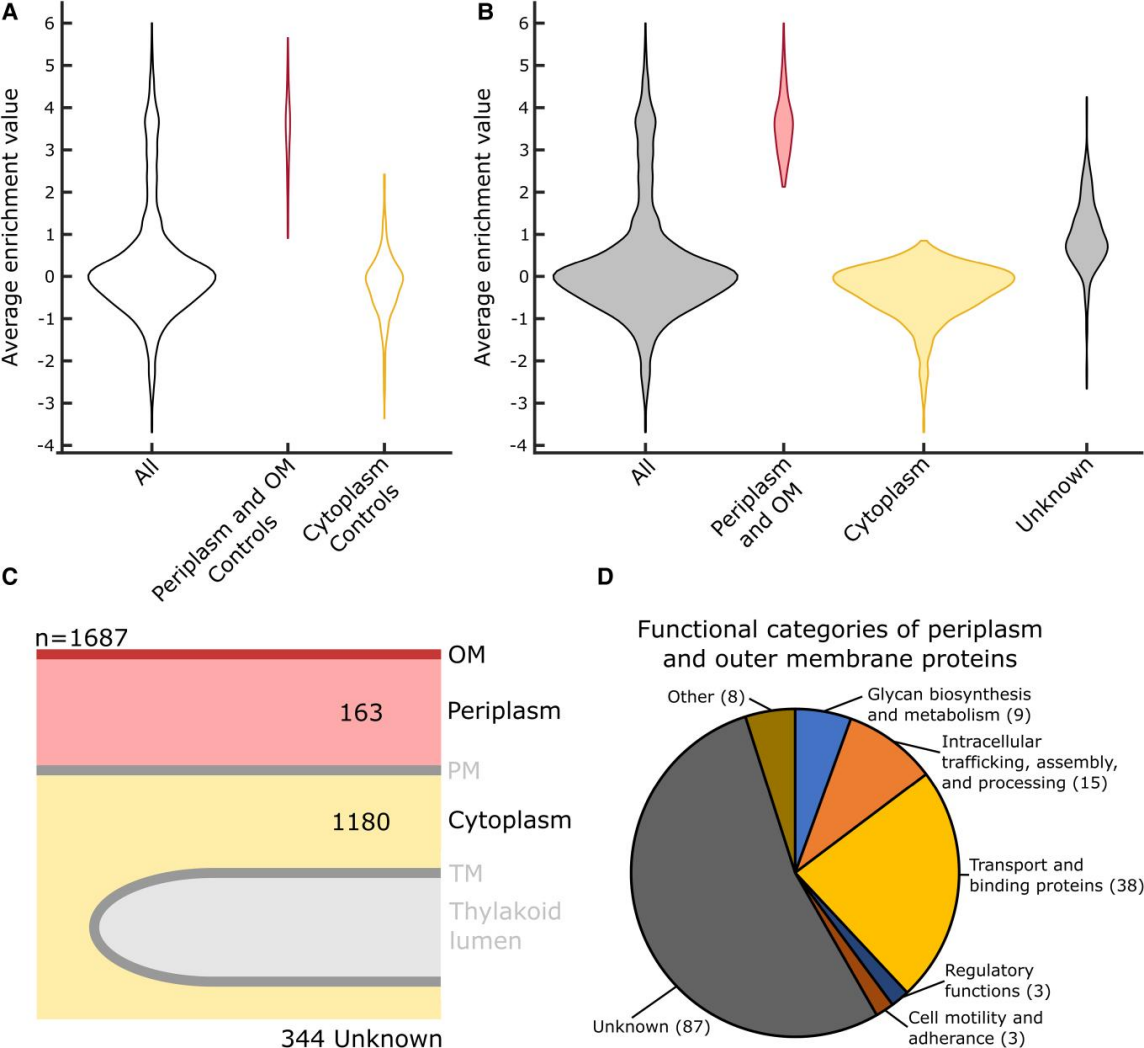
P-OM analysis. **A)** A violin plot showing distribution of average enrichment value (log_2_ [periplasm/cytoplasm]) for all proteins (*n* = 1,687) and control proteins with known localizations in the P-OM (*n* = 65) and cytoplasm (*n* = 381). Size of the violins is normalized by the number of samples in each category. **B)** A violin plot showing distribution of average enrichment values (log_2_ [periplasm/cytoplasm]) for proteins (*n* = 1,687) localized to the P-OM (*n* = 163), cytoplasm (*n* = 1180), as well as proteins that were unable to be localized in this analysis (Unknown) (*n* = 344). Size of the violins is normalized by the number of samples in each category. **C)** An infographic of the protein localizations determined in the analysis of the P-OM proteome. **D)** The functional categories of proteins localized to the P-OM proteome in this analysis.

TMT enrichment values calculated using the ratios of periplasmic APEX2 protein fusions (A1097 and A1761) and cytoplasmic APEX2 protein fusions (GFP and CpcB) were compared with known periplasm and outer membrane (P-OM) protein controls (Supplementary Table S2) to determine localization (Fig. 5A). Mean enrichment values for this analysis are shown in Fig. 5B. One hundred sixty-three proteins were localized to the P-OM, 1,180 proteins were localized to the cytoplasm, and 344 proteins were unable to be confidently localized in this specific analysis (Fig. 5C). Eighty-six percent (56/65) of P-OM control proteins detected by MS in this study were identified in the P-OM proteome. An additional 37 P-OM control proteins were not detected in this study, bringing the coverage of the P-OM proteome to 55% (56/102). This lower coverage is somewhat expected, as the proteins expressed in the periplasm change significantly in different environmental conditions (Fulda et al. 2000).

Proteins in the P-OM proteome have expected characteristics of those localized to the periplasm, OM, extracellular space, and/or inner membrane (Table 2). Eighty-three percent of the P-OM proteome possesses a predicted signal sequence or at least one transmembrane helix. Furthermore, 57% of P-OM proteome homologs have been previously localized to the periplasm, OM, or extracellular space in PCC 6803 (Fulda et al. 2000; Sergeyenko and Los 2000; Huang et al. 2004; Klinkert et al. 2004; Kurian et al. 2006b; Rajalahti et al. 2007; Aldridge et al. 2008 ; Trautner and Vermaas 2013; Gao et al. 2014a; Cheregi et al. 2015; Sacharz et al. 2015; Dong et al. 2016; Oliveira et al. 2016; Wang et al. 2022). An additional 34% of P-OM proteome homologs have been localized to the inner membrane in PCC 6803 (Supplementary Table S3) (Zak et al. 1999 , 2001; Huang et al. 2002, 2006; Zhang et al. 2004, 2009; Pisareva et al. 2007, 2011; Rajalahti et al. 2007; Xu et al. 2008; Boehm et al. 2009; Schultze et al. 2009 ; Wegener et al. 2011; Li et al. 2012 ; Roberts et al. 2012; Liberton et al. 2016; Selão et al. 2016; Zhu et al. 2016; Baers et al. 2019; Wang et al. 2022). The functional categories of the P-OM proteome were also analyzed (Fig. 5D). Like the thylakoid lumen proteome, the largest functional category was “proteins with an unknown function” (87, 53%). The 2nd largest category is “transport and binding proteins” with 38 (23%) proteins. The periplasm, OM, and inner membrane are known to be involved in nutrient uptake, so transport and binding proteins are expected to be a major functional category (Nicolaisen et al. 2009; Liberton et al. 2016; Kowata et al. 2017; Miller and Salama 2018). The third largest category was “intracellular trafficking, assembly, and processing,” like the thylakoid lumen. This category contained 15 (9%) proteins, including the PSII assembly proteins PratA and A2745, a homolog of Sll0606 in PCC 6803. The 4th largest functional category of P-OM proteins, with 9 (6%) proteins, was “glycan biosynthesis and metabolism,” which included proteins involved in cell wall synthesis and maintenance.

**Table 2.** P-OM proteome information

Category	Number	%
Previous periplasm, OM, or extracellular localizations (PCC 6803 homologs only, *n* = 96)	55	57.3^a^
Previous periplasm, OM, extracellular, or inner membrane localizations (PCC 6803 homologs only, *n* = 96)	88	91.7^a^
Possess a signal sequence or transmembrane helix	136	83.4
Known P-OM proteins (65 detected by MS)	56	86.2^b^

^a^The percentage of proteins with homologs in PCC 6803 (96 total).

^b^The percentage of P-OM control proteins detected by MS (65 total) in the P-OM proteome.

### Cytoplasm proteome analysis

**Figure 6.**
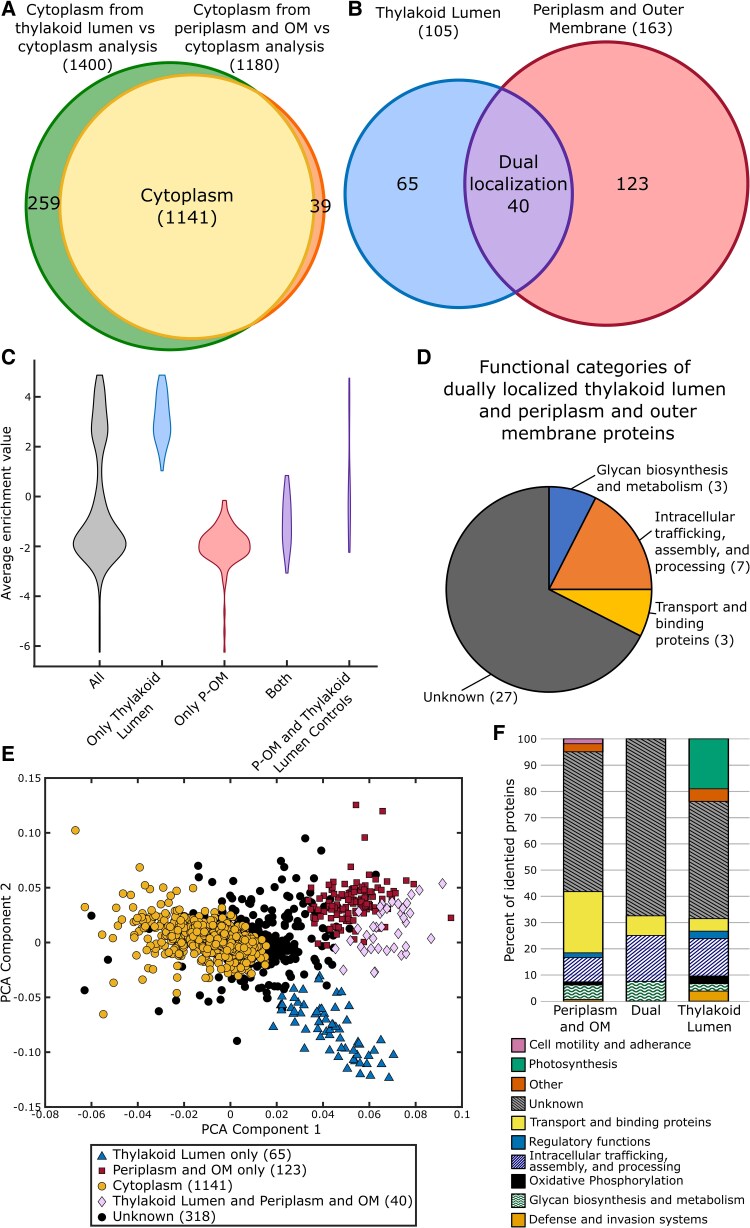
Dually localized proteins were identified using proteomics of specific cellular regions. **A)** A Venn diagram of the proteins with a final localization in the cytoplasm after analysis of the thylakoid lumen and the P-OM proteomes. **B)** A Venn diagram of protein dually localized to both the thylakoid lumen and the P-OM proteomes. **C)** A violin plot of the distribution of the average enrichment (log_2_ [thylakoid lumen/periplasm]) for the proteins (*n* = 228) in only the thylakoid lumen (*n* = 65), only the P-OM (*n* = 123), and both the thylakoid lumen and P-OM (*n* = 40) proteomes. The distribution of average enrichment scores for proteins that acted as controls for both the thylakoid lumen and P-OM analyses (*n* = 13) are on the far right. Size of the violins is normalized by the number of samples in each category. **D)** A pie chart of the functional categories of proteins dually localized to the thylakoid lumen and the P-OM. **E)** A PCA plot showing clusters of proteins that were identified as localized to the cytoplasm, thylakoid lumen, P-OM, and both the thylakoid lumen and P-OM and proteins that were unable to be confidently localized in this study. The key is below the plot. **F)** A bar chart showing the relative abundance of the functional categories of proteins identified in the thylakoid lumen (*n* = 105), P-OM (*n* = 163), and dually localized proteins (*n* = 40).

The cytoplasmic proteome was assembled from and intersection of the cytoplasm-localized proteins identified in the analysis of the thylakoid lumen proteome (PsbQ and A2695 compared with GFP and CpcB) and the P-OM proteome (A1097 and A1761 compared with GFP and CpcB) (Fig. 6A). Of the 1,141 proteins in the cytoplasm, only 2% possessed a predicted signal sequence and 6% possessed a predicted transmembrane helix. Eighty-six percent (328/381) of previously known cytoplasmic proteins detected by MS were identified in our cytoplasmic proteome. The known cytoplasmic list contained an additional 57 proteins that were not detected with MS in this study, so the estimated coverage of the cytoplasm proteome is 75%. The cytoplasmic proteins are distributed more evenly among functional categories than the thylakoid lumen or the P-OM proteomes (Supplementary Table S3). The largest functional category was “proteins of unknown function” (287, 25%), followed by “translation” (95, 8%), “nucleic acid metabolism” (81, 7%), “energy metabolism” (including photosynthesis and oxidative phosphorylation) (75, 7%), “amino acid metabolism” (71, 6%), “prosthetic groups, cofactors, and carriers” (70, 6%), “intracellular trafficking, processing, and assembly” (65, 6%), “carbohydrate metabolism” (61, 5%), “signal transduction” (58, 5%), and “defense and invasion systems” (55, 5%). Additional functional categories making up <5% of the cytoplasmic proteome were also present.

### Thylakoid lumen and P-OM proteome dual localization

Forty proteins were identified in the thylakoid lumen and the P-OM proteome and represent proteins with dual localizations (Fig. 6B). Interestingly, several dually localized control proteins present in the thylakoid lumen cluster in Fig. 3B were localized only to the thylakoid lumen in this study. This may be due to lower coverage of the P-OM proteome or to protein localization differences between PCC 6803 and PCC 7002. The dually localized proteins tended to be biotinylated by the thylakoid lumen and periplasm localized APEX2 a similar amount, with a bias toward more protein present in the periplasm than in the thylakoid lumen (Fig. 6C). Ninety-five percent of dually localized proteins possessed a predicted signal sequence or a transmembrane helix. Of the 23 dually localized proteins that have a homolog in PCC 6803, 18 (78%) have previously been localized to both the thylakoid lumen or membrane and the periplasm, extracellular space, OM, or inner membrane. The majority of the dually localized proteins were functionally categorized as “proteins with unknown function” (27, 68%) (Fig. 6D). The next largest category was “intracellular trafficking, assembly, and processing” with 7 proteins (15%). This category includes the PSII assembly factor PratA, 5 peptidases (HhoA, YmxG, PqqE, CtpB, and Prc), and PpiB, a folding chaperone. Three proteins involved in transport and binding and 3 proteins involved in glycan biosynthesis and metabolism were also dually localized. Dually localized proteins appear to cluster between the thylakoid lumen and periplasm clusters on a PCA plot (Fig. 6E).

Several patterns arose during the comparison of the relative abundance of the functional categories of proteins localized to the thylakoid lumen, P-OM, and the dually localized thylakoid lumen and P-OM proteins (Fig. 6F). In PCC 7002, the thylakoid lumen participates in photosynthesis, while the P-OM does not. However, the presence of Pitt and the Sll0606 homolog in the P-OM suggests that the P-OM plays a role in the early assembly of photosynthetic complexes. As expected, the P-OM proteome has a higher number of proteins involved in canonical P-OM roles, including transport and binding, cell motility and adherence, and glycan biosynthesis and metabolism. Proteins involved in “intracellular trafficking, assembly, and processing” made up a higher proportion of identified proteins in the thylakoid lumen and dually localized proteins, suggesting that these cellular regions are involved in the assembly of large protein complexes localized to the TM. This finding supports the hypothesis that PSII assembly begins in the inner membrane/periplasm and utilizes specialized assembly regions like the PratA-defined membrane prior to completion in the TM/lumen (Rengstl et al. 2011; Heinz et al. 2016 ). Our identification of proteins shared by both compartments adds further support to this hypothesis and provides a list of proteins that may be involved in nascent assembly of the TM.

### Calculation of signal sequence characteristics

**Figure 7.**
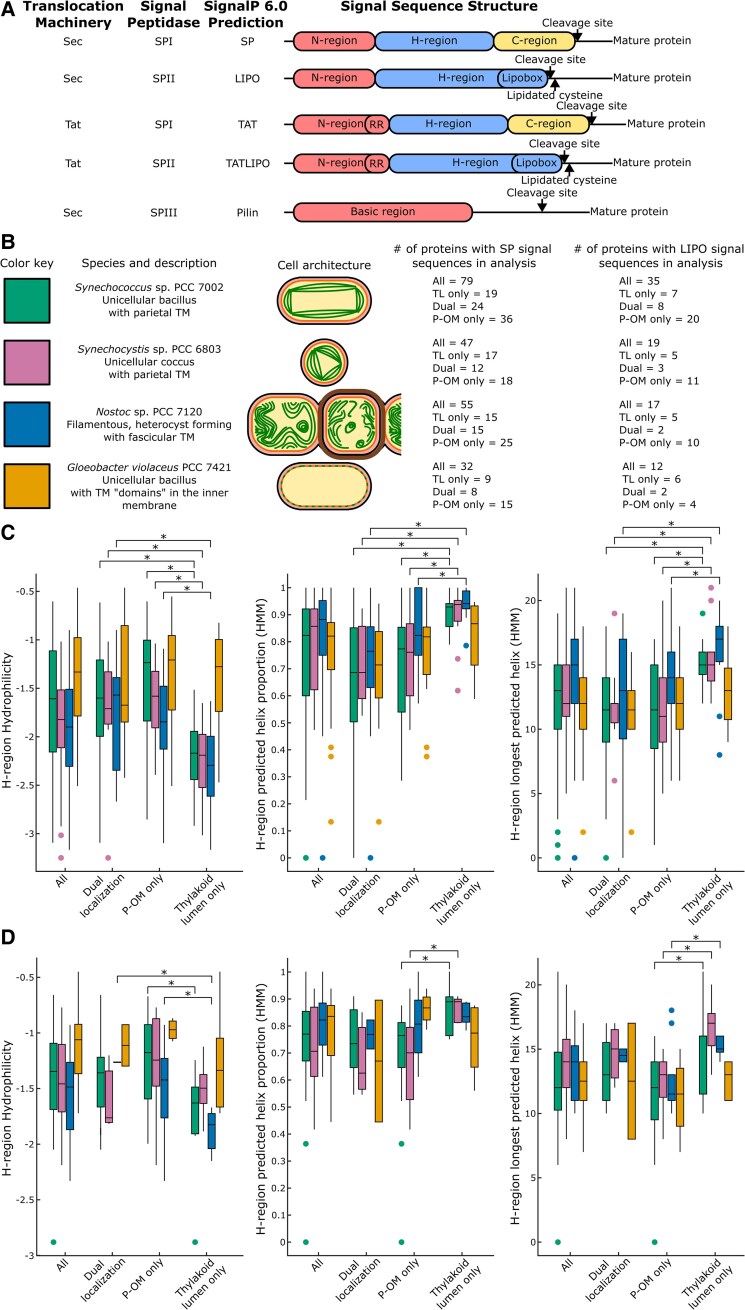
Sec-translocated signal sequence characteristics correlated with localization in cyanobacteria. **A)** Summary of signal sequence structures predicted by SignalP 6.0. **B)** Summary of the cyanobacteria cell architecture, TM characteristics, and signal sequences used in the analysis. TL only were thylakoid lumen proteins that were not localized to the P-OM in PCC 7002. P-OM only were proteins localized to the P-OM, but not the thylakoid lumen in PCC 7002. Dual localization proteins were localized to the thylakoid lumen and P-OM in PCC 7002. For the other species of cyanobacteria, the signal sequences used for analysis are signal sequences from best reciprocal BLAST homologs of proteins identified in the thylakoid lumen proteome and/or the P-OM proteome of PCC 7002 in this study. **C** and **D)** Tukey boxplots of H-region characteristics of signal sequences in different cellular localizations in different species. The center line represents the median, the box limits represent the upper (25th percentile) and lower (75th percentile) quartiles, outliers are >1.5 × the difference between the upper and lower quartiles, also known as the interquartile range, away from their nearest quartile, and the whiskers represent either (i) the minimum or maximum observed value when there are no outliers or (ii) the outlier limit (distance of 1.5 × the interquartile range away from the nearest quartile). Localization is listed on the *x* axis, and different species are in different colors (key in 7B) with the order in each localization cluster (All, dual localization, P-OM only, and thylakoid lumen only) from left to right as follows: PCC 7002, PCC 6803, PCC 7120, and PCC 7421. The sample size represented by each box and whisker plot is listed in **B)**. Significant differences (*P* < 0.05) between specific localizations within a species are marked by *. **C)** Displays characteristics for SP signal sequences or signal sequences predicted to be translocated across the membrane using Sec machinery and cleaved by SPI. **D)** Displays characteristics for LIPO signal sequences or signal sequences predicted to be translocated across the membrane using Sec machinery and cleaved by SPII.

The proteins with predicted signal sequences in the thylakoid lumen, P-OM, or both proteomes were analyzed to identify signal sequence characteristics that correlate with localization. In addition to the 2 translocation pathways Sec and Tat, cyanobacteria possess Type I, Type II, and Type III signal peptidases, which cleave the signal sequence from the processed protein. The different types of signal sequences have different structures (Fig. 7A) (Teufel et al. 2022), so we hypothesized that different types of signal sequences might have different properties that correlate with localization. Therefore, we sought to identify signal sequence characteristics correlated with localization in specific signal sequence types.

The presence and type of signal sequences were predicted with SignalP 6.0 (Teufel et al. 2022). There are 5 categories of signal sequences predicted by SignalP 6.0; 3 Sec-translocated categories: SP (SPI cleaved), LIPO (SPII cleaved), and Pilin (SPIII cleaved); and 2 Tat-translocated categories: TAT (SPI cleaved) and TATLIPO (SPII cleaved). The signal peptidase cleavage site and the N- and H-regions of the SP, LIPO, TAT, and TATLIPO signal sequences were also predicted by SignalP 6.0, along with the C-regions of SP and TAT signal sequences and the twin arginine motif in TAT and TATLIPO signal sequences. The N-region of signal sequences is the positively charged N-terminus of the signal sequence of variable length that averages around 5 aa (von Heijne 1990; Owji et al. 2018). The H-region of signal sequences is a hydrophobic region typically 7 to 15 aa long, although it can be longer (von Heijne 1990; Owji et al. 2018). The C-region is a 3 to 7 aa region following the H-region and containing the SPI cleavage motif (von Heijne 1990; Owji et al. 2018). The lipobox cleavage sequence of lipoproteins is considered an extension of the H-region, so they lack a C-region. For each protein localized to the thylakoid lumen and/or the P-OM proteome with a predicted signal sequence, descriptive characteristics were calculated for the entire signal sequence and the N-, H-, and C-regions. See Supplementary Table S4 for the values used for signal sequence analysis of PCC 7002.

### Analysis of signal sequence characteristics associated with localization in PCC 7002

*t*-tests were performed to determine if each characteristic calculated from signal sequences between proteins is correlated with localization (Supplementary Tables S4 and S5). Unfortunately, low numbers of TAT (*n* = 6), TATLIPO (*n* = 4), and PILIN (*n* = 1) proteins in the thylakoid lumen or P-OM proteomes prevented the identification of signal sequence characteristics significantly correlated with localization. However, there were enough SP (*n* = 79) and LIPO (*n* = 35) signal sequences to identify characteristics correlated with localization (Fig. 7B). When dually localized thylakoid lumen and P-OM proteins were excluded from the analysis, 105 signal sequence characteristics were significantly different (*P* < 0.05) between SP proteins localized to the thylakoid lumen and the P-OM (Supplementary Table S5). Several highly significant (*P* < 10^−5^) characteristics are shown in Fig. 7C. The highly significant values included 10 characteristics of the H-region and a single characteristic of the entire signal sequence. Therefore, the H-region contains the strongest differentiating characteristics that were measured in this analysis. These highly significant characteristics included the mean hydrophilicity and GRAVY score of the H-region, which measure a similar phenomenon but are anti-correlated. Many highly significant values were secondary structure characteristics of the H-region, including the longest predicted alpha helix, the alpha helix proportion, and the mean prediction confidence of the alpha helix. H-regions of thylakoid lumen SP signal sequences tended to be more hydrophobic and have a long structured alpha helix, in contrast to the H-regions of the P-OM signal sequences.

There were 66 statistically significant (*P* < 0.05) characteristics of LIPO signal sequences correlated with localization to the thylakoid lumen or P-OM when dually localized proteins were excluded (Supplementary Table S5). The signal sequence characteristics with the strongest significance values (*P* < 0.001) were autocorrelation of the hydrophilicity between every 4th amino acid of both the entire signal sequence and the H-region, the GRAVY score of the H-region, and the mean prediction confidence of alpha helix of the entire sequence. Similar to the SP signal sequences, LIPO signal sequences of thylakoid lumen proteins tended to be more hydrophobic and possess a longer alpha helix than periplasmic proteins (Fig. 7D).

### Analysis of signal sequence characteristics associated with localization in PCC 6803, PCC 7120, and PCC 7421

To determine if signal sequence characteristics correlated with localization in PCC 7002 were conserved among other cyanobacteria species, we analyzed signal sequences of the best reciprocal blast homologs of PCC 7002 thylakoid lumen and/or P-OM proteomes in PCC 6803, *Nostoc* sp. PCC 7120 (PCC 7120), and *Gloeobacter violac*eus (PCC 7421) (Fig. 7B; Supplementary Tables S6 and S7). PCC 6803 is a model unicellular cyanobacterium, PCC 7120 is a model heterocyst forming filamentous cyanobacteria, and PCC 7421 is a cyanobacteria lacking distinct TMs and a thylakoid lumen, with specialized domains in its inner membrane where the light reactions of photosynthesis occur instead. The homologs of PCC 7002 proteins were assumed to have the same cellular localization in their respective strains, with the exception of PCC 7421, where the homologs of PCC 7002 thylakoid lumen proteins are expected to localize in the periplasm near the photosynthetic inner membrane domains. Signal sequence characteristics were calculated for each thylakoid lumen and/or P-OM homolog with a predicted signal sequence, and *t*-tests were used to identify characteristics significantly correlated with localization for both SP and LIPO signal sequences separately (Supplementary Table S7). In PCC 6803, 66 characteristics of SP signal sequences (*n* = 47) were statistically significant (*P* < 0.05) (Supplementary Table S8). Similar to PCC 7002 SP signal sequences, PCC 6803 SP thylakoid lumen signal sequences are more structured, particularly in alpha helixes, and the H-region is more hydrophobic than periplasmic signal sequences (Fig. 7C). There were 40 characteristics of LIPO signal sequences in PCC 6803 (*n* = 19) that were significantly associated with localization (*P* < 0.05) (Supplementary Table S8). Similar to PCC 7002 LIPO signal sequences, LIPO thylakoid lumen signal sequences are more structured, particularly in alpha helixes, than periplasmic signal sequences in PCC 6803 (Fig. 7D). The hydrophobicity measures of LIPO signal sequences in PCC 6803 were not significantly correlated with localization. When dually localized proteins were excluded, there were 70 characteristics of PCC 7120 SP signal sequences (*n* = 55) and 26 characteristics of PCC 7120 LIPO signal sequences (*n* = 17) that were significantly different (*P* < 0.05) between thylakoid lumen and periplasmic proteins (Supplementary Table S8). Similar to PCC 7002 SP and LIPO signal sequences, the PCC 7120 thylakoid lumen signal sequences had H-regions that were more alpha-helical and hydrophobic than periplasm signal sequences (Fig. 7, C and D). In PCC 7421, there were 25 SP (*n* = 32) and 17 LIPO (*n* = 12) signal sequence characteristics that were significantly different between homologs to PCC 7002 proteins localized to the thylakoid lumen and PCC 7002 homologs localized to the periplasm when the dually localized PCC 7002 homologs were excluded (Supplementary Table S8). Unlike PCC 7002, there were not significantly different structural or hydrophobicity measures between the PCC 7421 homologs of PCC 7002 proteins localized to the thylakoid lumen and PCC 7421 homologs of PCC 7002 proteins localized to the periplasm (Fig. 7, C and D).

## Discussion

This study demonstrates the activity of APEX2 in multiple cellular environments, including the cytoplasm, the low pH thylakoid lumen, and the oxidizing periplasmic space. Furthermore, proteomes generated from different APEX2 fusion proteins in the same membrane-bound compartment maintained similar profiles while remaining distinct from other membrane-bound compartments (Figs. 2 and 3; Supplementary Figs. S2 and S4). The proteomics data from APEX2 localized to the cytoplasm and thylakoid lumen had low correlation. There was a higher correlation between APEX2 localized to the periplasm and cytoplasm (Fig. 3A), which is likely a result of APEX2 targeted to the periplasm with bait proteins containing a Tat signal sequence. The periplasmic APEX2 fusion proteins fold completely in the cytoplasm before translocation into the periplasm using the Tat signal sequence. Because of this, some of the periplasmic A1097 and A1761-APEX2 fusion proteins will be folded and functional in the cytoplasm, resulting in some biotinylation of cytoplasmic proteins, which increases the correlation between periplasmic and cytoplasmic samples. The thylakoid lumenal PsbQ and A2695-APEX2 fusion proteins have a Sec signal sequence, which translocates unfolded proteins, often cotranslationally (Frain et al. 2016). These proteins are less likely to exist in a folded form in the cytoplasm than in the Tat-translocated periplasmic APEX2 fusion proteins, explaining the lower correlation between thylakoid lumen samples and cytoplasmic samples. Despite the high correlation between proteomes from APEX2 localized to the same membrane-bound compartment, there is differential labeling of specific proteins that the fusion proteins are expected to associate with more closely (Supplementary Fig. S6 and Text). Therefore, the APEX2 labeling technique can be used to identify potential interaction partners of specific proteins of interest, particularly in the cytoplasm. In fact, this technique has been used to discover members of protein interaction networks in PCC 6803 (Santos-Merino et al. 2024). APEX2 labeling could be especially useful in the discovery of weakly associated proteins that may not be identified using copurification techniques. This paper already indicates that there are subdomains within the periplasm and cytoplasm based on the unique but similar proteomes detected using different bait proteins in these compartments.

Of the 1,687 proteins identified in this study, 1,416 were able to be confidently localized. Additional machine learning analysis of the proximity-based proteomics data gave similar localization results to the analysis method used in this paper but were unable to identify proteins dually localized in the periplasm and thylakoid lumen (Supplementary Fig. S7 and Text). Two hundred seventy-one proteins were unable to be confidently localized in this study. Many of these proteins were membrane proteins, where APEX2 biotinylation is expected to occur on both sides of the membrane, making localization difficult when comparing the thylakoid lumen or P-OM labeling patterns with the cytoplasm. However, by comparing the thylakoid lumen and periplasm APEX2 labeling patterns, many integral membrane proteins were able to be localized to the TM or the inner membrane (Supplementary Fig. S8 and Text). Despite this additional analysis, APEX2 labeling and analysis was able to localize a much greater proportion of soluble proteins than membrane proteins. Unlike traditional membrane purification techniques, which are able to determine the localization of more integral membrane proteins than the APEX2 technique used here, APEX2 is a technique that works best for soluble proteins within a membrane-bound space. In the future, APEX2 proteomics could be used in parallel with membrane purification techniques to localize proteins to both membranes and membrane-bound compartments.

The proteomes identified in this study were analyzed to determine the functional roles of different parts of the cyanobacteria cell. In agreement with previous cyanobacteria proteomics studies, we found that the proteome of TM included proteins involved in photosynthesis and energy generation, while the inner membrane proteome included proteins important for metabolite transport, cell motility, and cell wall synthesis and maintenance (Supplementary Fig. S8) (Liberton et al. 2016; Baers et al. 2019). The thylakoid lumen proteome consists of proteins involved in several cellular processes, including photosynthesis and the assembly and processing of the photosynthetic complexes and other proteins in the thylakoid lumen and TM. The P-OM proteome is involved in metabolite transport and binding, cell wall maintenance, and cell motility and adherence.

Interestingly, our study revealed many proteins of unknown function in both the thylakoid lumen and the P-OM proteomes. Determining the functions of these proteins localized specifically to the thylakoid lumen or P-OM could reveal additional roles for these cellular regions, adding to our understanding of cyanobacteria cell biology. Furthermore, the proteins dually localized to the thylakoid lumen and the periplasm provide further evidence of a cellular region with characteristics of the periplasm and the thylakoid lumen involved in TM biogenesis and PSII assembly. Of the 40 dually localized proteins reported in this study, 27 have an unknown function, making this region of particular interest for future studies.

The proteomes of the thylakoid lumen and the P-OM described in this study allowed signal sequence characteristics to be correlated with localization. For signal sequences of Sec-translocated proteins cleaved by SPI or SPII in PCC 7002, a more hydrophobic H-region with an alpha helix secondary structure is correlated with localization in the thylakoid lumen (Fig. 7). More hydrophilic H-regions with less alpha helix secondary structures are associated with P-OM localization. Homologs of proteins localized to the thylakoid lumen or P-OM proteomes in this study were identified in PCC 6803, PCC 7120, and PCC 7421 and their signal sequences were analyzed. PCC 6803 and PCC 7120 represent diverse model cyanobacteria species, while PCC 7421 represents a cyanobacteria outgroup without an internalized TM. Similar to PCC 7002, increased hydrophobicity and alpha-helical secondary structure in the H-region of signal sequences in PCC 6803 and PCC 7120 was correlated with thylakoid lumen localization for proteins translocated by Sec machinery and cleaved by SPI. PCC 6803 and PCC 7120 proteins translocated by Sec and cleaved by SPII, if not significant, trend toward a hydrophobic alpha-helical H-region association with thylakoid lumen localization. Therefore, for Sec-translocated SPI cleaved (SP) signal sequences the correlation between thylakoid lumen localization and a hydrophobic alpha-helical H-region of the signal sequence is conserved across multiple species of cyanobacteria. This is also likely true for Sec-translocated SPII cleaved (LIPO) signal sequences, but the smaller sample size of LIPO signal sequences makes reaching statistical significance more difficult. As expected, PCC 7421 signal sequences did not have H-region hydrophobicity or alpha-helical secondary structure correlations with localization of the homologs in PCC 7002 thylakoid lumen. All translocated proteins in PCC 7421 are translocated to the periplasmic space instead of the thylakoid lumen (Fig. 7).

Interestingly, the signal sequence characteristics correlated with localization in the thylakoid lumen in this study were not reported in a previous publication by Rajalahti et al. (2007), a study that identified volume and polarizability of amino acids at specific positions in the signal sequence, as well as volume of certain amino acids in the N-terminal region of the processed proteins as factors important for localization of soluble proteins to the thylakoid lumen or periplasm. We note that Rajalahti et al. did not analyze the secondary structure of these signal sequences, explaining some of the differences between the results of their study and ours. However, the largest difference with this study was that Rajalahti et al. (2007) lacked a comprehensive dataset of proteins in the thylakoid lumen, as there was no technique to purify thylakoid lumen proteins from cyanobacteria at that time. We also note that they analyzed less signal sequences, grouped different types of signal sequences together (SP, TAT, and LIPO), and did not analyze specific regions (N-, H-, or C-) of signal sequences, which likely decreased the strength of correlations. Thus, their previous study did not report hydrophobicity as an important factor for sorting between the periplasm and thylakoid lumen.

The sensitivity and specificity of the signal sequence analysis is limited due to the software used. While SignalP 6.0 is one of the most accurate signal sequence prediction softwares with a high specificity, a known limitation is that the classification has a lower sensitivity compared to its specificity. In other words, the software is more likely to provide false-negative predictions (a protein with a signal sequence is classified as not having a signal sequence) a one than to predict a false positive (FP; a protein without a signal sequence classified as having a signal sequence) (Teufel et al. 2022). Consequently, there are likely additional proteins possessing signal sequences in the thylakoid lumen and P-OM proteomes that incorrectly classified and subsequently excluded from the signal sequence analysis. Furthermore, the accuracy of secondary structure prediction also limits the analysis. The 3-state (α-helix, β-strand, and coil) accuracy of JPred4 is 81.5% (Drozdetskiy et al. 2015). While the accuracy of SignalP 6.0 and JPred4 is sufficient to reveal overall trends, additional research and analysis will need to be done to verify the signal sequences predicted in this study. Supplemental experiments are needed to verify that the signal sequence characteristics associated with localization are causal, although conservation of the pattern across cyanobacterial species with separate thylakoid and inner membrane systems supports this hypothesis.

There are many mechanisms that could result in more hydrophobic and alpha-helical H-region of their Sec signal sequences targeting proteins to the thylakoid lumen. For example, the TM and inner membrane have different membrane compositions, and signal sequences could be interacting directly with the membrane (Omata and Murata 1983). Additionally, the H-region of the signal sequences could be directly interacting with membranes or the SecYEG translocon, a trimeric Sec protein-conducting channel composed of SecY, SecE, and SecG, which may have slightly different conformations in different membranes, allowing for more specific interactions with the H-region of signal sequences. Cyanobacteria typically have only one copy of SecYEG in their genome, which excludes the possibility that different SecYEGs are localized in each membrane and have specificity for certain signal sequences (Russo and Zedler 2020). Furthermore, Sec machinery has been localized to both the TM and inner membrane in cyanobacteria (Nakai et al. 1993).

Alternatively, there could be chaperone proteins acting similar to SecA or SecB in cyanobacteria that may be involved in targeting proteins to either the TM or inner membrane. SecA is an ATPase important for translocation across the membrane that can interact indirectly through chaperone proteins or directly with the signal sequence of a translocated protein to bring the translocated protein to the SecYEG machinery (Cranford-Smith and Huber 2018). SecB is a protein that binds unfolded proteins that will be translocated (Frain et al. 2016). Cyanobacteria typically have one copy of SecA in the genome and do not have a copy of SecB (Russo and Zedler 2020). Therefore, in cyanobacteria, there are not multiple copies of SecA differentiating between signal sequences and associating with specific membranes. While cyanobacteria do not have SecB, they do have multiple copies of DnaJ and DnaK proteins, which can partially recover wild type phenotypes in *secB* knockouts in *Escherichia coli* (Wild et al. 1992). Certain DnaJ and DnaK proteins are important for cell growth and survival in different environments in cyanobacteria (Nimura et al. 2001; Düppre et al. 2011). Furthermore, the DnaK proteins localized in Supplementary Fig. S1 have different localizations, which could result from the different roles that different DnaK proteins may play in protein targeting. Further research is needed to identify the mechanism by which signal sequences are sensed and used to determine whether a protein should be translocated across the TM or inner membrane. Because cyanobacteria and chloroplasts share a common ancestor, the mechanism used to sort proteins in cyanobacteria may be conserved in chloroplasts.

Signal sequence characteristics may also work in tandem with other protein targeting systems in cyanobacteria. For example, certain membrane proteins are targeted to their proper membrane system through localization of their mRNA to specific regions of the PM or TM (Mahbub et al. 2020; Mahbub and Mullineaux 2023). In these cases, RNA-binding chaperone proteins direct mRNA localization to the correct membrane (Mahbub et al. 2020; Wang et al. 2024). Localization of mRNAs corresponding to soluble thylakoid lumen or periplasmic proteins has not yet been reported in cyanobacteria. Further research is needed to determine what role mRNA localization and RNA-binding chaperone proteins play in localization of soluble proteins translocated across the TM or inner membrane.

Altogether, this study represents a comprehensive proteomic analysis of a cyanobacterial cell, providing a compartmentalome that reveals the subcellular localization of both membrane and soluble proteins and insights into the sorting mechanisms. We expect the database generated in this study to be a valuable resource to the scientific community.

## Materials and methods

### Creation of PCC 7002 strains

All genes were amplified from PCC 7002 with the exception of GFP, APEX2, and the V5 epitope. APEX2 was amplified from a plasmid gifted to us by Alice Ting (Addgene plasmid # 72558; http://n2t.net/addgene:72558; RRID: Addgene_72558). Plasmids were assembled using Gibson Assembly (Gibson et al. 2009) with p*_ccmK2_* as the promoter (Cameron et al. 2013), kanamycin resistance for selection, and a C-terminal tag of a short linker sequence followed by either superfolder GFP-V5 (GFP-V5) or APEX2-V5 on a PCC 7002 gene. GlpK was used as a neutral site for incorporation into the PCC 7002 genome (Ruffing et al. 2016). The Gibson reactions were transformed into DH5α *E. coli* or CopyCutter EPI400 *E. coli* when the plasmid was toxic in DH5α, and minipreps of liquid cultures started from single colonies were performed to collect plasmid. Plasmid was transformed into PCC 7002 (Stevens and Porter 1980), and colonies containing the desired insert were serially passaged in the presence of antibiotic until segregated.

### Wide-field fluorescence microscopy

Wide-field microscopy was performed as reported in Dahlgren et al. (2021). Briefly, cells were spotted onto an agar pad (A+ w/v 1% agarose) and placed onto a microscope slide. Cells were imaged with a Nikon TiE inverted wide-field microscope. A standard Cy5 emission filter set (Nikon) (collection bandwidth is 665 to 715 nm) utilizing excitation from a 640-nm LED light source (SpectraX) at 50% power was used to image chlorophyll fluorescence of TMs. A standard GFP emission filter set (Nikon) (collection bandwidth is 515 to 555 nm) utilizing excitation from a 470-nm LED light source (SpectraX) at 50% power was used to image GFP localization or anti-V5 immunofluorescence.

### Proximity-based biotinylation of proteins by APEX2

Biotinylation of proteins was performed using a modified protocol from Hung et al. (2016) and Hwang and Espenshade (2016), which was optimized for PCC 7002 (Dahlgren et al. 2021). Briefly, 50 mL cultures of PCC 7002 strains were grown in A+ media (Stevens et al. 1973) in air at 37 °C with a light intensity of 185 *µ*mol photons m^−2^ s^−1^ for 2 d to an OD_730_ of about 0.5. The culture was pelleted at 4,300 × *g* for 10 min at 4 °C. The supernatant was poured off and cells were resuspended in 4 mL A+ medium with 1.5 mm BP (biotin-phenol) and transferred to a 6-well plate. Six-well plates were incubated shaking in air at 37 °C with a light intensity of 185 *µ*mol photons m^−2^ s^−1^ for 30 min. Samples were then pelleted in a 1.5 mL tube and resuspended in 1 mL phosphate buffered saline (PBS) pH 7.8 (Bio-Rad) containing 1.5 mm BP. Ten microliters of 100 mm H_2_O_2_ were added, and cells were mixed by inverting for 30 s before pelleting for 30 s. Supernatant was removed, and cells were resuspended in quencher solution (PBS with 10 mm sodium ascorbate, 5 mm trolox, and 10 mm sodium azide) and pelleted to wash the cells 3 times. Cell pellets were frozen at − 80 °C for storage and to facilitate cell lysis.

### Cell lysis

Cells were lysed by bead beating in radioimmunoprecipitation assay lysis buffer as previously reported (Dahlgren et al. 2021).

### Protein concentration measurement

The protein concentration of cell lysate was quantified using the Pierce 660 nm Protein Assay (Thermo Fisher Scientific).

### Immunoblotting

Proteins were separated on a 10% SDS-PAGE gel, and immunoblots were performed following the protocol from Green and Sambrook (2012). Protein was transferred to a nitrocellulose membrane or a PVDF membrane if fluorescent secondary antibodies were used, since PVDF membranes have a lower background fluorescence than nitrocellulose membranes. To monitor transfer efficiency to blots, a Ponceau stain was performed before membranes were blocked. Membranes were incubated with primary anti-V5 (Invitrogen, cat. no. R960-25) antibodies or streptavidin-HRP (horseradish peroxidase) (Life Technologies, cat. no. R960-25). Membranes probed for V5 were then incubated with a secondary antibody conjugated to HRP or AlexaFluor 488 (Thermo Fisher Scientific, cat. no. A-11008 or cat. no. 31460). Membranes were visualized using chemiluminescence after exposure to the Clarity Western ECL substrate (Bio-Rad) or fluorescence.

### Immunofluorescence

PCC 7002 cells were fixed using a protocol modified from Buddelmeijer et al. (2013). Briefly, 1.5 mL of PCC 7002 at an OD_730_ of 0.3 to 0.4 were pelleted, washed with 500 *µ*L PBS, and resuspended in 500 *µ*L PBS. To fix cells, the cells were incubated in 0.04% glutaraldehyde and 2.8% formaldehyde for 15 min standing at room temperature with no added light. Cells were then washed with PBS 4 times before storage at 4 °C for up to a month before imaging. After fixation, cells were permeabilized and labeling for immunofluorescence was performed according to Buddelmeijer et al. (2013). Briefly, cells were incubated in 0.1% Triton X-100 for 45 min at room temperature to permeabilize membranes. Cells were washed 3 times with PBS, and then cell walls were digested by incubating cells for 45 min with 100 *µ*g/mL lysozyme in 5 mm EDTA in PBS. Cells were again washed with PBS 3 times. Next, cells were blocked with blocking buffer (PBS containing 0.5% bovine serum albumin) for 1 h at room temperature and then washed 3 times with PBS. Primary antibody (anti-V5, Invitrogen, cat. no. R960-25) at a 1:500 dilution in blocking buffer was incubated with cells for 1 h. Then, cells were washed 3 times in PBS with 0.05% Tween-20. Cells were incubated for 1 h with secondary antibody (anti-mouse AlexaFluor 488 [Thermo Fisher, cat. no. 31460]) at a 1:200 dilution in blocking buffer with no added light. Cells were washed 3 more times with PBS containing 0.05% Tween-20 before washing once more with PBS. Finally, cells were resuspended in 150 *µ*L PBS.

### Silver staining

Proteins were separated on a 10% SDS-PAGE gel and stained using the short silver nitrate staining protocol as described (Chevallet et al. 2006).

### Purification of biotinylated proteins

Biotinylated proteins were purified on streptavidin beads as reported in Dahlgren et al. (2021) with modifications to the ratio of lysate to beads.

### Elution of biotinylated proteins for silver stain and immunoblot analysis

Beads were denatured at 98 °C for 10 min in 30 *µ*L of elution buffer (3× Laemmli buffer, 2 mm biotin, and 20 mm DTT) to elute biotinylated proteins. The eluate was collected and diluted with 60 *µ*L of water to run on gels.

### Beta-barrel prediction

The prediction of beta-barrel OM proteins was done using PRED-TMBB2 (Tsirigos et al. 2016).

### Transmembrane Helix prediction

To predict if a protein had transmembrane helices, all proteins in the UniProt reference proteome for PCC 7002 and PCC 6803 were analyzed using the TMHMM Server v. 2.0 (Krogh et al. 2001).

### Signal sequence prediction

To predict if a protein had a signal sequence and the cut site to remove the signal sequence, all proteins in the UniProt reference proteome for PCC 7002 and PCC 6803 were analyzed with SignalP-5.0 (Almagro Armenteros et al. 2019) using both the Gram-positive and Gram-negative bacterial options. For the analysis of signal sequence characteristics, signal sequence prediction was performed using SignalP-6.0 (Teufel et al. 2022) using the “Other” organism option and “Slow” model mode on the UniProt reference proteomes for PCC 7002, PCC 6803, PCC 7120, and PCC 7421. The N-, H-, and C-regions of the signal sequence, as well as the signal peptidase cut site, were predicted using SignalP-6.0.

### Sample preparation for MS

Biotinylated proteins enriched on streptavidin beads from 3 replicates each of strains GFP-APEX2-V5, CpcB-APEX2-V5, A2695-APEX2-V5, PsbQ-APEX2-V5, A1097-APEX2-V5, and A1761-APEX2-V5 were eluted, reduced, and alkylated using 3× Laemmli sample loading buffer (187.5 mm Tris, 6% [w/v] SDS, 20 mm DTT, 30% [v/v] glycerol, 0.015% [w/v] bromophenol blue, and 2 mm biotin). Biotin-enriched samples were reduced and alkylated with the addition of 5% (w/v) SDS, 10 mm TCEP (tris(2-carboxyethyl)phosphine), 40 mm 2-chloroacetamide, and 50 mm Tris-HCl, pH 8.5 boiled for 10 min and then incubated shaking at 2,000 rpm at 37 °C for 30 min. Purified proteins were digested using the SP3 method (Hughes et al. 2014). Briefly, 200 *µ*g carboxylate-functionalized speedbeads (Cytiva Life Sciences) were added followed by the addition of acetonitrile (ACN) to 80% (v/v) inducing binding to the beads. The beads were washed twice with 80% (v/v) ethanol and twice with 100% ACN. Proteins were digested in 50 mm Tris-HCl, pH 8.5, with 0.5 *µ*g Lys-C/Trypsin (Promega) and incubated at 37 °C overnight. Tryptic peptides were desalted using a Waters M-class UPLC with a photodiode array detector and a custom fabricated 0.5 × 150 mm UChrom rpC18 1.8 *µ*m 120 Å (nanolcms) column. Cleaned-up peptides were then dried in a speedvac vacuum centrifuge and stored at −20 °C. Peptides were labeled with the Tandem Mass Tags (TMTpro) 18-plex (Thermo Fisher Scientific) according to the manufacturer's instructions. Briefly, peptides were suspended in 0.1 m triethylammonium bicarbonate, and each TMT label was added in ACN and incubated for 1 h at ambient. TMTpro Labeling reactions were quenched with the addition of hydroxylamine and incubated for 15 min at ambient and then combined. The multiplexed sample was then desalted using a 1-cc (10 mg) Waters HLB Oasis cartridge according to the manufacturer's instructions and dried in a speedvac vacuum centrifuge.

### MS and data analysis

TMTpro-labeled peptides were suspended in 3% (v/v) ACN and 0.1% (v/v) trifluoroacetic acid and directly injected onto a reversed-phase charged surface hybrid column rpC18 1.7 *µ*m, 130 Å, 75 × 250 mm M-class column (Waters), using an Ultimate 3000 nanoUPLC (Thermo Fisher Scientific). Peptides were eluted at 300 nL/min with a gradient from 4% to 25% ACN in 120 min and then to 40% ACN in 5 min and detected using a Q-Exactive HF-X mass spectrometer (Thermo Fisher Scientific). Precursor mass spectra (MS1) were acquired at a resolution of 120,000 from 350 to 1500 m/z with an automatic gain control (AGC) target of 3E6 and a maximum injection time of 50 ms. Precursor peptide ion isolation width for MS2 fragment scans was 0.7 m/z with a 0.2-m/z isolation offset, and the top 15 most intense ions were sequenced. All MS2 spectra were acquired at a resolution of 45,000 with higher energy collision dissociation at 30% normalized collision energy. An AGC target of 1E5 and 120 ms maximum injection time was used. Dynamic exclusion was set for 20 s with a mass tolerance of ±10 ppm. Rawfiles were searched against the UniProt *Synechococcus* sp. database UP000001688 downloaded on June 4, 2022 containing 3179 genes using MaxQuant v.2.0.3.0. Cysteine carbamidomethylation and the TMTPro label were considered fixed modifications, while methionine oxidation and protein N-terminal acetylation were searched as variable modifications. All peptide and protein identifications were thresholded at a 1% false discovery rate. The MS proteomics data have been deposited in the ProteomeXchange Consortium via the PRIDE partner repository with the dataset identifier PXD058004. Contaminants and proteins with <2 spectral counts, as well as proteins lacking TMT reporter ion intensity values, were filtered out of the MS data. Therefore 1,687 proteins were used for quantitative proteomic analysis.

### Calculation of enrichment values between samples

Cyclic Loess normalization was used to normalize the MS samples. To perform the normalization, the MS data were first logarithmically transformed using the equation ln (value + 1). Next, the normalizeCyclicLoess command from the *limma* package of Bioconductor was used to normalize the logarithmically transformed dataset in R (Ritchie et al. 2015). The normalized values were then back-transformed using the equation e^normalized value. The back-transformed normalized values are listed in Supplementary Table S3. Enrichment ratios were calculated for a protein using the equation log_2_([sample 1 normalized value + 1]/[sample 2 normalized value + 1]). Enrichment values were calculated for every protein (1,687 total) for each pairwise sample comparison. The means of the enrichment values calculated from the 3 replicates of a strain compared with the 3 replicates of a second strain are listed in Supplementary Table S3.

### Determining protein localization from enrichment values

A modified version of the analysis method described by Dahlgren et al. (2021) and Hung et al. (2016) was used to determine protein localization . Analysis 1 was used to identify proteins localized to the thylakoid lumen. In this analysis, each lumenal replicate (PsbQ-APEX2 and A2695-APEX2) was paired with each cytoplasmic (GFP-APEX2 and CpcB-APEX2) replicates for a total of 36 comparisons. For each single comparison (e.g. PsbQ-APEX2 Replicate 1 and GFP-APEX2 Replicate 1) the proteins were organized from highest to lowest enrichment value. Then, the true positive (TP) and FPs were assigned according to proteins known or expected to localize to the regions of interest. The TP proteins (thylakoid lumen proteins) contained 3 categories of proteins. The 1st category is PCC 7002 homologs of proteins previously identified as thylakoid lumen localized in multiple PCC 6803 studies (Kashino et al. 2002, 2006; Rajalahti et al. 2007; Aldridge et al. 2008; Schultze et al. 2009; Wegener et al. 2011; Heinz et al. 2016; Xiao et al. 2022) where both the PCC 6803 and PCC 7002 homologs possess a predicted signal sequence and/or one predicted transmembrane helix. The 2nd category is PCC 7002 homologs of PCC 6803 proteins that were localized to the thylakoid lumen in only one study (Kashino et al. 2002, 2006; Rajalahti et al. 2007; Aldridge et al. 2008; Schultze et al. 2009; Wegener et al. 2011; Heinz et al. 2016; Xiao et al. 2022) where both the PCC 7002 and PCC 6803 homologs possess a signal sequence and/or one transmembrane helix, and the PCC 7002 homolog was also found in the thylakoid lumen by Dahlgren et al. (2021). The final category of TP proteins are proteins localized to the thylakoid lumen of PCC 7002 during APEX2 localization studies in Supplementary Figs. S1 and S2. The FP proteins (cytoplasmic proteins) were PCC 7002 homologs of PCC 6803 proteins that were found in the soluble proteome in at least 4 studies (Choi et al. 2000; Simon et al. 2002; Lindahl and Florencio 2003; Gan et al. 2005; Fulda et al. 2006; Pérez-Pérez et al. 2006; Slabas et al. 2006; Kurian et al. 2006a, 2006b; Mata-Cabana et al. 2007; Gao et al. 2009, 2014b, 2015; Pandhal et al. 2009; Rowland et al. 2011; Fuszard et al. 2013; Mehta et al. 2014; Mikkat et al. 2014; Plohnke et al. 2015; Baers et al. 2019; Wang et al. 2022), had no predicted signal sequence or transmembrane helix in PCC 7002 or PCC 6803, and were found in 1 or less studies of the TM (Zak et al. 1999, 2001; Wang et al. 2000; Fulda et al. 2002; Ohkawa et al. 2002; Herranen et al. 2004; Zhang et al. 2004; Srivastava et al. 2005; Komenda et al. 2006; Pisareva et al. 2007, 2011; Xu et al. 2008; Boehm et al. 2009; Agarwal et al. 2010; Rowland et al. 2010; Rengstl et al. 2011; Roberts et al. 2012; Sacharz et al. 2015; Liberton et al. 2016; Selão et al. 2016; Zhu et al. 2016; Baers et al. 2019; Wang et al. 2022), thylakoid lumen (Kashino et al. 2002, 2006; Rajalahti et al. 2007; Aldridge et al. 2008; Schultze et al. 2009; Wegener et al. 2011; Heinz et al. 2016; Xiao et al. 2022), periplasm (Fulda et al. 2000; Klinkert et al. 2004; Kurian et al. 2006b; Rajalahti et al. 2007; Aldridge et al. 2008; Sacharz et al. 2015; Dong et al. 2016; Wang et al. 2022), or inner membrane (Zak et al. 1999, 2001; Huang et al. 2002, 2006; Zhang et al. 2004, 2009; Pisareva et al. 2007, 2011; Rajalahti et al. 2007; Xu et al. 2008; Boehm et al. 2009; Schultze et al. 2009; Wegener et al. 2011; Li et al. 2012; Roberts et al. 2012; Liberton et al. 2016; Selão et al. 2016; Zhu et al. 2016; Baers et al. 2019; Wang et al. 2022). The TP and FP proteins can be found in Supplementary Table S2. For each protein in a comparison, the TP rate (TPR) and the FP rate (FPR) were calculated. The TPR for a specific protein was the number of TP proteins with an enrichment value greater than or equal to the enrichment of the specific protein divided by the total number of TP proteins found in the experiment. The FPR for a specific protein was the number of FP proteins with an enrichment value greater than or equal to the enrichment of the specific protein divided by the total number of FP proteins. The cutoff for each sample was the enrichment with the greatest difference between the TPR and FPR value. If a protein was above the cutoff in 35 or more of the 36 analyses, it was declared as localized to the thylakoid lumen. Furthermore, a reverse comparison using the enrichment values from the cytoplasmic samples over the thylakoid lumen samples and using the cytoplasmic proteins as the TP list and thylakoid lumen proteins as the FP list was performed. If a protein was above the cutoff in 35 or more of the 36 comparisons, it was declared as localized to the cytoplasm in the thylakoid lumen/cytoplasm comparison. Proteins that did not meet the cutoff for either thylakoid lumen or cytoplasm in this analysis were not able to be localized and categorized as unknown.

Analysis 2 used a technique similar to the method used to identify thylakoid lumen proteins in the P-OM. In this analysis, each periplasmic replicate (A1097-APEX2 and A1761-APEX2) was paired with each cytoplasmic replicate (GFP-APEX2 and CpcB-APEX2) for a total of 36 comparisons. The TP proteins (P-OM proteins) again included 3 categories of proteins. The 1st category is OM PCC 7002 proteins, which were defined as proteins with a predicted beta-barrel fold possessing a signal sequence or a single transmembrane helix. The 2nd category is proteins localized to the periplasm in PCC 7002 in Supplementary Fig. S1, including A0860, A1761, and A1097. The last category of proteins in the P-OM TP list is PCC 7002 homologs of PCC 6803 proteins possessing a signal sequence or only 1 transmembrane helix that was secreted or localized to the OM or periplasm in one or more previous studies (Fulda et al. 2000; Sergeyenko and Los 2000; Huang et al. 2004; Klinkert et al. 2004 ; Kurian et al. 2006b; Rajalahti et al. 2007; Aldridge et al. 2008; Trautner and Vermaas 2013; Gao et al. 2014a; Cheregi et al. 2015; Sacharz et al. 2015; Dong et al. 2016; Oliveira et al. 2016 ; Wang et al. 2022). The P-OM TP list is in Supplementary Table S2. The FP list was the same cytoplasmic proteins used for the thylakoid lumen analysis. The TPR and FPR were calculated for each protein, and cutoff was selected to maximize the difference between the TPR and FPR values. For a protein to be defined as localized to the P-OM in this dataset, the protein must be above the cutoff in 35 or more of the 36 comparisons. Similar to the thylakoid lumen analysis, a reverse analysis was performed to determine proteins localized to the cytoplasm in the periplasm/cytoplasm comparison. Proteins above the cutoff in 35 or more of the 36 comparisons were defined as cytoplasmic in this analysis. Proteins that did not meet the cutoff for either thylakoid lumen or cytoplasm localization in these analyses were not able to be localized and categorized as unknown. However, if any of the cytoplasm or unknown proteins were categorized as localized to the thylakoid lumen in the thylakoid lumen analysis, that localization took precedence. Similarly, any protein identified as cytoplasmic or unknown in the thylakoid lumen analysis and identified as P-OM localized in the periplasm analysis was considered localized to the P-OM. Forty proteins were localized to both the thylakoid lumen and P-OM. Proteins localized to the cytoplasm in both the P-OM and thylakoid lumen analyses were localized to the cytoplasm. The remaining proteins without a thylakoid lumen, P-OM, or cytoplasm localization were categorized as proteins with an unknown localization.

### Signal sequence analysis

Proteins with a localization predicted in the thylakoid lumen and/or P-OM were used for signal sequence analysis. Each protein was resubmitted to SignalP-6.0, and the slow version was used to better predict the signal sequence regions. Only proteins with SP, LIPO, TAT, or TATLIPO signal sequences with a length >5 predicted by SignalP-6.0 were used in the signal sequence analysis. The best reciprocal BLAST homolog of each 7002 protein localized to the lumen and/or P-OM in this paper was determined for the following cyanobacterial species: PCC 6803, PCC 7120, and PCC 7421. The localization of the homologs in other species was assumed to be the same as the localization identified in this study of the 7002 homolog. Structural prediction on the signal sequences was performed using JPred4 (Drozdetskiy et al. 2015). Other parameters of the entire signal sequences, as well as the N-, H-, and C-regions of the signal sequences as determined by SignalP-6.0 were calculated with the ProtParam module of the SeqUtils package of Bio package in Biopython, including the length, number, and proportion of each of the 20 amino acids, GRAVY score, isoelectric point, molecular weight, and the charge at pH 7, 8, and 9 (Cock et al. 2009). Additional values describing signal sequences, including the number of positively charged amino acids and number of negatively charged amino acids, were calculated using custom code. Furthermore, custom code was used to calculate the mean properties the signal sequence and the N-, H-, and C-regions using the descriptor scales z_1_, z_2_, and z_3_ for each amino acid as described by Hellberg et al. (1987). The z_1_, z_2_, and z_3_ scales correspond to hydrophobicity/hydrophilicity, side-chain volume/bulk, and polarizability/electronic properties, respectively. To compare patterns in amino acid properties within signal sequences between sequences of different lengths, the z_1_, z_2_, and z_3_ scales were used to perform auto- and cross-correlation studies of the signal sequences and the H-region with custom code using equations from Sjöström et al. (1995). Furthermore, custom code was used to calculate structural values for each of the signal sequences and the N-, H-, and C-regions from the JPred4 data. From these data, the maximum amino acid length of predicted alpha helixes, beta sheets, or both alpha helixes and beta sheets, and the number of amino acids and proportion of the signal sequence in predicted alpha helixes, beta sheets, or neither secondary structure were calculated. The mean confidence of alpha helix, beta sheet, and neither secondary structure prediction was also calculated. Furthermore, the predictions of amino acids buried at specific solubilities from JPred4 were used to determine the longest buried region, and the number and proportion of amino acids buried in a signal sequence were calculated. Statistical analyses involved performing student *t*-tests comparing signal sequences characteristics of differently localized proteins in each species and were carried out in Python 3.10 using custom code.

### Accession numbers

Sequence data from this article can be found in the GenBank/EMBL data libraries under accession number_CP000951.1.

## Supplementary Material

kiaf186_Supplementary_Data

## Data Availability

The majority of data underlying this article are available in the article and in its online Supplementary material. The database of PCC 6803 localizations and a summary of the protein localizations determined in this study are also available at https://dahlgren-lab.github.io/cyano-compartmentalome-data/. The MS data are available in the ProteomeXchange Consortium via the PRIDE partner repository with the dataset identifier PXD058004. The UniProt *Synechococcus* sp. database UP000001688 contains the protein sequences used to identify proteins from the MS data. Sequence data from this article can be found in the GenBank/EMBL data libraries under accession number CP000951.1.

## Supplementary data

The following materials are available in the online version of this article.

**Supplementary Figure S1.** Localization of GFP- and APEX2-tagged proteins.

**Supplementary Figure S2.** Biotinylation patterns of APEX2 fusion proteins localized to different membrane-bound compartments.

**Supplementary Figure S3.** Immunoblots and gels of biotinylated protein samples used for MS.

**Supplementary Figure S4.** PCA plot shows clustering of samples within the same membrane-bound compartment.

**Supplementary Figure S5.** Comparison between the thylakoid lumen proteome to other recent thylakoid lumen proteomes.

**Supplementary Figure S6.** Differential labeling between APEX2 fusion proteins within membrane-bound compartments.

**Supplementary Figure S7.** Machine learning protein localization analysis.

**Supplementary Figure S8.** Membrane proteomics analysis.

**Supplementary Table S1.** PCC 6803 localization database.

**Supplementary Table S2.** Control proteins with known localizations.

**Supplementary Table S3.** Proximity-based proteomics localization results.

**Supplementary Table S4.** PCC 7002 signal sequence analysis.

**Supplementary Table S5.** PCC 7002 statistically significant signal sequence characteristics.

**Supplementary Table S6.** Homologs in PCC 6803, PCC 7120, and PCC 7421.

**Supplementary Table S7.** PCC 6803, PCC 7120, and PCC 7421 signal sequence analysis.

**Supplementary Table S8.** PCC 6803, PCC 7120, and PCC 7421 statistically significant signal sequence characteristics.

## Dive Curated Terms

The following phenotypic, genotypic, and functional terms are of significance to the work described in this paper:

peptides CHEBI: CHEBI:16670
