# Supplementary material for: Spatial proteomics reveals signal sequence characteristics correlated with localization in cyanobacteria
Source: Plant Physiol. 2025 Aug 6;198(4):kiaf186. doi: 10.1093/plphys/kiaf186 (PMC12341915; doi:10.1093/plphys/kiaf186)
Supplement: kiaf186_Supplementary_Data [file kiaf186_supplementary_data.zip › Supplementary compartmentalome text final.pdf]

Supplemental Text

Supplemental Results:

Targeting APEX2 to different membrane-bound compartment

The localization of protein candidates used to target APEX2 were verified with C-terminal GFP fusions (Supplemental Fig. S1A). In addition to GFP alone, the localization of a number of cytoplasmic proteins fused to GFP was confirmed with fluorescence, including ApcA, ApcB, CpcA, CpcB, DnaK1, DnaK2, DnaK3, DnaK4, Fur, FusA, Ppx, RbcL, RplA, TrpE, Vipp1, and Ycf39. GFP fused to the Tat signal sequence-possessing proteins A1097, A1761, and A0860 had clear periplasmic localization. We observed GFP fluorescence in the periplasm only when GFP was fused to a protein with a Tat signal sequence in PCC 7002. Tat signal sequences translocate proteins across the membrane in a folded state, so although the superfolder GFP used in this study folds in the periplasm of other bacteria, such as *E. coli* when fused to a Sec signal sequence (Dinh and Bernhardt, 2011), it is possible that superfolder GFP does not fold in the periplasm of PCC 7002 when fused to a Sec signal sequence. GFP fluorescence in the thylakoid lumen of PCC 7002 was generally low, likely due to GFP's 80% decrease in fluorescence at pH 5, the pH of the thylakoid lumen in PCC 7002 (Belkin et al., 1987; Stoddard and Rolland, 2019). However, a low GFP signal associated with chlorophyll fluorescence was observed in GFP fusions to PsbQ, A2695, A0101, and Ycf48 (Dahlgren et al., 2021).

Next, localization of APEX2 protein fusions was verified with immunofluorescence against a V5 epitope fused to the C-terminus of APEX2 (Supplemental Fig. S1B). The V5 epitope does not contain electron-rich amino acids commonly biotinylated by biotin phenol radicals (Rhee et al., 2013), allowing for detection of the epitope in samples were APEX2 dependent biotinylation has been performed. Localization of APEX2 fusion proteins to the cytoplasm was confirmed for CpcB, DnaK3, Rpe, and RplA using this method. CpcB, Rpe, and RplA were closely associated with the thylakoid membranes. Using immunofluorescence, APEX2 fusions to A1097 and A1761 were localized to the periplasm. APEX2 fusions to PsbQ, A2695, PetJ, Psb32, PsbU, and Ycf48 were localized to the thylakoid lumen using immunofluorescence.

Functionality of APEX2 and localization of proteins was confirmed by performing APEX2 dependent biotinylation and probing cell lysate for biotinylated proteins on an immunoblot. The functionality of 35 APEX2 fusion proteins were tested (Supplemental Fig. 2C and Fig. S2), and only 7 fusion proteins (PilB, ApcA, CpcA, Fur, DnaK4, Ppx, and A2577) did not biotinylate proteins at a detectable level via immunoblot. Therefore, 80% of APEX2 fusion proteins were able to biotinylate proteins in the conditions tested.

APEX2 fusion proteins localized to the cytoplasm, thylakoid lumen, and periplasm have different labeling patterns (Supplemental Fig. S2). Proteins with a thylakoid lumen localization have a prominent band of biotinylated proteins at 33 kDa. This band is observed in a biotinylation immunoblot of APEX2 fusions to PsbU, A0101, A2695, PsbQ, PsbV, Ycf48, PetJ, Psb32, PpiB, YmxG, YtfC, CrtU, and PratA. Proteins with a periplasmic localization have prominent bands at 50 and 52 kDa. The periplasmic labeling pattern was observed in APEX2 fusions to A0860, A1097, and A1761. Cytoplasmic biotinylation patterns lack the prominent bands present in the thylakoid lumen and periplasm. This pattern was observed in GFP, ApcB, CpcB, Ycf39, DnaK1, DnaK2, DnaK3, Rpe, FusA, RplA, and Vipp1 APEX2 fusion proteins. The unique biotinylation pattern observed in RbcL-APEX2-V5 is likely due to its localization in carboxysomes and procarboxysomes (Cameron et al., 2013). The large band present at ~55 kDa in streptavidin blot likely represents RbcL (52 kDa).

Visualizing mass spectrometry results with PCA

To visualize similarity between replicates and different APEX2 fusion proteins, a principal component analysis using the normalized TMT values of each protein was performed. The components explaining the largest differences were plotted in Supplemental Fig. S4. Replicates of each APEX2 fusion protein tended to cluster together. All three replicates from both APEX2 fusion proteins localized to the cytoplasm, thylakoid lumen, or periplasm formed 3 larger clusters.

Differential labeling of proteins by different APEX2 fusions in the same membrane-bound compartment

An analysis of the proteomics data of the two differently localized cytoplasmic APEX2 constructs was able to identify proteins specifically enriched in the GFP-APEX2 sample or the CpcB-APEX2 sample (Supplemental Fig. S6A). Statistically significant proteins with a $\log_2$ (enrichment of GFP-APEX2 over CpcB-APEX2) over 0.5 included carboxysome shell proteins CcmK1 and CcmK2, and carboxysomal carbonic anhydrase CcaA. Additionally, DNA-associated proteins such as homologs of the plasmid partitioning protein ParB, transcription factors such as LexA, and RNA polymerase subunits RpoA, RpoB, RpoZ, and SigC were also enriched in the GFP-APEX2 cytoplasmic sample. In the CpcB-APEX2 sample, there was a statistically significant enrichment for phycobilisome proteins ApcA, CpcA, CpcD, and CpcG, as well as subunits of thylakoid membrane protein complexes PSI (PsaE and PsaF), ATP synthase (AtpC, AtpD, AtpF, and AtpG), NADH dehydrogenase (NdhJ and NdhO), and ferredoxin-NADP+ reductase PetH, and the twin-arginine translocation subunit TatA. Additionally, PSI assembly factors Ycf37, RubA, and Ycf3 were enriched in the CpcB-APEX2 cytoplasmic sample. The differences in enriched proteins between the GFP-APEX2 sample and the CpcB-APEX2 sample correspond to the differences in localization of the two protein fusions. GFP-APEX2 is localized away from the thylakoid membranes in the cytoplasm, resulting in more labeling of DNA and carboxysome associated proteins. In contrast, the APEX2 fusion to the phycobilisome subunit CpcB labeled phycobilisome proteins and membrane-associated proteins. The differences in labeling between the two samples highlight the ability of APEX2 to determine local proteomes within a specific compartment in cyanobacteria.

In contrast the cytoplasmic APEX2 constructs, the lumen localized PsbQ-APEX2 and A2695-APEX2 labeling had very few proteins with statistically significant proteins with log2 enrichment change greater than 0.5 (Supplemental Fig. S6B). We speculate that this may be due to the small size of the thylakoid lumen. The thylakoid lumen is only 15 nm wide in PCC 6803 (Liberton et al., 2013), and likely has a similar width in PCC 7002. The width of the thylakoid lumen is similar to the 10-20 nm labeling radius of APEX2, preventing significant differences between proteins localized within this small compartment (Rhee et al., 2013). The small space in the thylakoid lumen could explain the lack of differential enrichment of proteins between the lumenal samples.

The periplasmic APEX2 constructs displayed differences in enrichment (Supplemental Fig. S6C). Out of the 31 proteins with a statistically significant $\log_2$ (enrichment) in A1761 greater than 1, 20 proteins were localized to the thylakoid lumen or thylakoid membrane in this study. It is possible that A1761 fused to APEX2 is more likely to be mislocalized to the thylakoid lumen than A1097. However, localization in the thylakoid lumen might be part of the normal A1761

expression, although it was not identified in the thylakoid lumen in this study. 196 statistically significant proteins were enriched by at least one-fold in A1097-APEX2 samples over A1761-APEX2 samples. An enrichment of inner membrane proteins is expected in the A1097-APEX2 enriched proteins, because A1097 is a lipoprotein associated with the inner membrane (Veit et al., 2016); in fact, 51 proteins localized to the inner membrane in this study (Supplemental Fig. S8) were enriched in A1097-APEX2 samples over A1761-APEX2 samples. Additionally, 22 proteins involved in transport or binding of metabolites localized to the P-OM in this study were enriched in A1097-APEX2.

Membrane protein localization analysis

The localization of many integral membrane proteins was unable to be determined in the through the analyses comparing the thylakoid lumen or periplasm samples to the cytoplasm. Integral membrane proteins can be labeled by an APEX2 fusion protein localized to either side of the membrane, and the majority of the 261 proteins with at least one predicted transmembrane helix were localized to the cytoplasm (73, 28%) or had an unknown localization (82, 31%). Therefore, to determine the localization of membrane proteins, enrichment values were calculated from the ratios of TMT values of the thylakoid lumen APEX2 fusion proteins (PsbQ and A2695) and periplasmic APEX2 fusion proteins (A1097 and A1761) for the integral membrane proteins and analyzed using lists of proteins with homologs in PCC 6803 known to localize to the thylakoid membrane or inner membrane (Zak et al., 1999; Wang et al., 2000; Zak et al., 2001; Fulda et al., 2002; Huang et al., 2002; Ohkawa et al., 2002; Herranen et al., 2004; Zhang et al., 2004; Srivastava et al., 2005; Huang et al., 2006; Komenda et al., 2006; Pisareva et al., 2007; Rajalahti et al., 2007; Xu et al., 2008; Boehm et al., 2009; Schultze et al., 2009; Zhang et al., 2009; Agarwal et al., 2010; Rowland et al., 2010; Pisareva et al., 2011; Rengstl et al., 2011; Wegener et al., 2011; Li et al., 2012; Roberts et al., 2012; Sacharz et al., 2015; Liberton et al., 2016; Selão et al., 2016; Zhu et al., 2016; Baers et al., 2019; Wang et al., 2022). In this analysis, proteins known to localize to the thylakoid membrane or the inner membrane were used as controls (Supplemental Fig. S8A). The distribution of mean enrichment values calculated from TMT values of the thylakoid lumen APEX2 fusion proteins over the periplasm APEX2 fusion proteins is shown in Supplemental Fig. S8B. There was substantial overlap between the enrichment values of the proteins with known localizations. The enrichment values of the proteins localized to the thylakoid membrane and the inner membrane in this study are shown in Supplemental Fig. S8C. In the analysis of the membrane proteins, 75 (29%) of proteins were localized to the thylakoid membrane, 77 (30%) of proteins were localized to the inner membrane, and 109 (42%) of proteins were not able to be localized to a specific membrane in this analysis (Supplemental Fig. S8D,E).

The thylakoid membrane proteome determined in this study consisted of 75 proteins with a predicted transmembrane helix. Of the 75 proteins with a known localization in the thylakoid membrane identified by mass spectrometry, 50 (67%) were localized to the thylakoid membrane in this study. The estimated coverage of the thylakoid membrane proteome is 47% because 50 out of 107 proteins with a known localization were identified in the thylakoid membrane. 58 (83%) of the 70 thylakoid membrane proteins with homologs in PCC 6803 have previously been localized to the thylakoid membrane (Zak et al., 1999; Wang et al., 2000; Zak et al., 2001; Fulda et al., 2002; Ohkawa et al., 2002; Herranen et al., 2004; Zhang et al., 2004; Srivastava et al., 2005; Komenda et al., 2006; Pisareva et al., 2007; Xu et al., 2008; Boehm et al., 2009; Agarwal et al., 2010; Rowland et al., 2010; Pisareva et al., 2011; Rengstl et al., 2011; Roberts et al., 2012;

Sacharz et al., 2015; Liberton et al., 2016; Selão et al., 2016; Zhu et al., 2016; Baers et al., 2019; Wang et al., 2022). Those 58 proteins were also all more often identified in studies of the thylakoid membrane than the inner membrane in PCC 6803 (Zak et al., 1999; Wang et al., 2000; Zak et al., 2001; Fulda et al., 2002; Huang et al., 2002; Ohkawa et al., 2002; Herranen et al., 2004; Zhang et al., 2004; Srivastava et al., 2005; Huang et al., 2006; Komenda et al., 2006; Pisareva et al., 2007; Rajalahti et al., 2007; Xu et al., 2008; Boehm et al., 2009; Schultze et al., 2009; Zhang et al., 2009; Agarwal et al., 2010; Rowland et al., 2010; Pisareva et al., 2011; Rengstl et al., 2011; Wegener et al., 2011; Li et al., 2012; Roberts et al., 2012; Sacharz et al., 2015; Liberton et al., 2016; Selão et al., 2016; Zhu et al., 2016; Baers et al., 2019; Wang et al., 2022). Homologs of five proteins in the thylakoid membrane proteome had only been localized to the inner membrane and not the thylakoid membrane in PCC 6803 and seven proteins have no specific localization data in PCC 6803.

The functional categories of the proteins present in the thylakoid membrane proteome were also analyzed (Supplemental Fig. S8F). 'Proteins with an unknown function' and 'photosynthesis' tied for the top functional category of thylakoid membrane proteins, with 20 proteins (27%) in each category. The PSI integral membrane subunits PsaA, PsaB, PsaF, PsaL, and PsaM were identified in the thylakoid membrane proteome. The PSII subunits PsbA, PsbB, PsbC, PsbE, and PsbH were also identified in the thylakoid membrane proteome, along with PsbQ, PsbV, PsbP, and PsbZ, which have a predicted signal sequence that overlaps with their predicted transmembrane helix. The localization of PsbD was unknown, likely due to its localization in the inner membrane during early steps of PSII assembly (Zak et al., 2001; Rengstl et al., 2011; Heinz et al., 2016). Cytochrome b_6f subunits, including PetA, PetB, PetC, and PetD were also identified in the thylakoid membrane proteome. Additionally, 5 (7%) proteins involved in 'oxidative phosphorylation', including the NADH dehydrogenase subunits NdhD2, NdhG, and NdhF and the ATP synthase subunits AtpF and AtpG were identified in the thylakoid membrane. The second largest functional category of thylakoid membrane proteins with 14 (19%) proteins is 'intracellular trafficking, assembly, and processing.' This category includes protein translocation subunits SecE, SecY, YidC, and TatA in addition to several folding chaperones and PSI and PSII assembly factors.

The inner membrane proteome determined in this study includes 77 predicted integral membrane proteins. Of the 57 proteins with a homolog in PCC 6803, 51 (89%) have previously been localized to the inner membrane (Zak et al., 1999; Wang et al., 2000; Zak et al., 2001; Fulda et al., 2002; Huang et al., 2002; Ohkawa et al., 2002; Herranen et al., 2004; Zhang et al., 2004; Srivastava et al., 2005; Huang et al., 2006; Komenda et al., 2006; Pisareva et al., 2007; Rajalahti et al., 2007; Xu et al., 2008; Boehm et al., 2009; Schultze et al., 2009; Zhang et al., 2009; Agarwal et al., 2010; Rowland et al., 2010; Pisareva et al., 2011; Rengstl et al., 2011; Wegener et al., 2011; Li et al., 2012; Roberts et al., 2012; Sacharz et al., 2015; Liberton et al., 2016; Selão et al., 2016; Zhu et al., 2016; Baers et al., 2019; Wang et al., 2022). An additional 3 proteins have homologs previously localized to the periplasm or outer membrane (Fulda et al., 2000; Klinkert et al., 2004; Kurian et al., 2006; Rajalahti et al., 2007; Aldridge et al., 2008; Sacharz et al., 2015; Dong et al., 2016; Wang et al., 2022). Only two proteins in the inner membrane proteome have been identified in more thylakoid membrane studies than inner membrane studies in PCC 6803 (Zak et al., 1999; Wang et al., 2000; Zak et al., 2001; Fulda et al., 2002; Huang et al., 2002; Ohkawa et al., 2002; Herranen et al., 2004; Zhang et al., 2004; Srivastava et al., 2005; Huang et al., 2006; Komenda et al., 2006; Pisareva et al., 2007; Rajalahti et al., 2007; Xu et al., 2008; Boehm et al., 2009; Schultze et al., 2009; Zhang et al., 2009; Agarwal et al., 2010; Rowland et

al., 2010; Pisareva et al., 2011; Rengstl et al., 2011; Wegener et al., 2011; Li et al., 2012; Roberts et al., 2012; Sacharz et al., 2015; Liberton et al., 2016; Selão et al., 2016; Zhu et al., 2016; Baers et al., 2019; Wang et al., 2022). The inner membrane proteome includes 46 (61%) of 76 proteins with a known inner membrane localization identified by mass spectrometry in this study. The coverage of the inner membrane proteome is estimated at 29%, because an additional 80 proteins with a known inner membrane localization were not detected by mass spectrometry in this study. Similar to the P-OM proteome, the significant changes occur in the inner membrane proteome in response to different environmental conditions (Huang et al., 2006; Zhang et al., 2009). This may partially explain the low coverage of the inner membrane, because only standard growth conditions were used in this study.

The functional categories of the inner membrane proteins were also analyzed (Supplemental Fig. S8F). The largest category was 'proteins with an unknown function' with 31 (40%) proteins. 'Transport and binding proteins' was the next largest functional category of inner membrane proteins with 18 (23%) proteins. 'Glycan biosynthesis and metabolism' was the third largest category with 8 (10%) of proteins. 'Glycan biosynthesis and metabolism' and 'transport and binding proteins' are expected to be major functional roles of proteins in the inner membrane, because the cell wall (composed of peptidoglycan) resides in the periplasmic space and metabolites required for cell growth must cross the inner membrane. Four (5%) protein involved in 'intracellular trafficking, assembly, and processing' were identified in the inner membrane, including the PSII assembly factor A2745, a homolog of sll0606 in PCC 6803. While one protein involved in oxidative phosphorylation (AtpH-II) was identified in the inner membrane, no photosynthetic proteins were.

109 predicted membrane proteins were unable to be localized to a specific membrane in this study. The majority of these proteins were localized to the cytoplasm proteome (61, 56%) by the other analyses performed in this study. A membrane protein that is exposed mostly on the cytoplasmic side would be labeled more strongly by cytoplasmic APEX2, leading to its localization in the cytoplasm in the analyses comparing the TMT values of thylakoid lumen or periplasm localized APEX2 to cytoplasmic APEX2. A lack of labeling on the periplasmic or thylakoid lumen exposed face of a integral membrane protein prevents differential labeling between periplasm and thylakoid lumen APEX2 fusion proteins, preventing localization to a specific membrane using this method. 35 (32%) of proteins with an unknown membrane localization were also unable to be localized to any of the soluble proteomes (thylakoid lumen, P-OM, or cytoplasm). These proteins with unknown localizations could represent the proteome of an intermediate membrane fraction, perhaps the PratA-defined membrane (Rengstl et al., 2011), with characteristics of the inner membrane and thylakoid membrane with exposure to the periplasm, thylakoid lumen, and cytoplasm. Furthermore, 10 proteins that were dually localized to the thylakoid lumen and the P-OM in this study were unable to be localized to a specific membrane, supporting their dual localization in both the inner membrane and the thylakoid membrane. The largest functional category of proteins with an unknown membrane localization is 'proteins with an unknown function' (48, 44%) (Supplemental Fig. S8F). The second largest functional category with 15 (14%) proteins was 'transport and binding proteins'. The next largest category was proteins involved in 'intracellular trafficking, assembly, and processing' with 12 (11%) proteins. These proteins included the PSII assembly protein PratA, the Sec translocation complex subunit SecD, a Tat translocation subunit, and three FtsH proteases. Interestingly, 6 (6%) proteins involved in 'oxidative phosphorylation', including AtpB, AtpFN, and four NADH dehydrogenase subunits, were unable to be localized to a specific membrane system in this study.

While the localization of many membrane proteins was unable to be specified in this study, 47 proteins that were unable to be localized to the thylakoid lumen, P-OM, or the cytoplasm were able to be localized to the thylakoid membrane or the inner membrane using the membrane protein analysis. Furthermore, an additional 12 predicted membrane proteins with a cytoplasmic localization were able to be localized to the thylakoid membrane or the inner membrane.

Proteins with Undefined Localizations
After determining the proteomes of the thylakoid lumen, P-OM, cytoplasm, thylakoid membrane, and inner membrane, 271 proteins without a localization remained (See Supplemental Fig. S8 for membrane proteome analysis and Fig. 6E). As predicted, this group of proteins with undefined localizations included several enzymes that interact with biotin and are therefore always purified on streptavidin beads. These proteins included the biotin carboxyl carrier protein AccB, biotin synthase BioB, and dethiobiotin synthetase BioD. There was also an enrichment of ribosomal proteins and other proteins involved in translation (31, 11%) in proteins with an unknown localization. Of the 20 phycobilisome proteins identified in this study, 7 had an unknown localization, including CpcB, one of the proteins used to localize APEX2 to the cytoplasm. An inability to localize many of the ribosomal and phycobilisome proteins is consistent with many previous proteomic studies in cyanobacteria, which commonly have the highly expressed phycobilisome and ribosome proteins present in purified subcellular fractions of cyanobacteria.

Machine learning analysis of protein localization
To demonstrate the robust nature of the proteomics dataset, two additional approaches to determine protein localization were performed. The first alternative method is unbiased k-means clustering. This algorithm divides data into clusters, grouping similar data points together. When k-means clustering was performed on mean enrichment values between each APEX2 fusion protein to obtain three clusters, the clusters closely match the thylakoid lumen, P-OM, and cytoplasm proteomes. (Supplemental Fig. S7A) Cluster 1 contains the entire cytoplasm proteome and the majority of proteins with an unknown localization in its 1394 proteins. Cluster 2 contains all but three proteins from the P-OM proteome, including 37 proteins identified in the thylakoid lumen and the P-OM, as well as a number of proteins with an unknown localization in its 206 proteins. The 87 proteins in cluster 3 contain a few unknown localization proteins and all thylakoid lumen proteins except the 37 proteins identified in both the thylakoid lumen and P-OM that were present in cluster 2.
A support vector machine (SVM) learning algorithm using mean enrichment values between each APEX2 fusion protein was trained on the proteins with known localizations, with proteins with expected dual localization to the thylakoid lumen and P-OM removed from the known localization list. This method resulted in protein localizations that correlate well with the localizations obtained from the analysis method used in this study (Supplemental Fig. S7B). The machine learning algorithm produced predictions for cytoplasm, P-OM, and proteins with a low confidence in prediction were classified as unknown. The SVM machine learning cytoplasm proteome of 1344 proteins contained 99% (1127) of the cytoplasm proteome and the majority of proteins with and unknown localization. The SVM machine learning thylakoid lumen proteome contained only 39 proteins from the thylakoid lumen proteome. The SVM machine learning P-OM proteome contained 209 proteins, including 91% (148) of the P-OM proteome and a few proteins with unknown localizations. The unknown localization category derived from proteins

that the SVM machine learning predicts of low confidence of localization included 129 proteins in the cytoplasm, thylakoid lumen, P-OM, dually localized thylakoid lumen and P-OM, and unknown localization proteomes. Overall, the high agreement on protein localization between the analyses using the enrichment values and proteins with known localizations, k-means clustering, and SVM machine learning supports the robustness of this dataset to describe the proteomes of the cytoplasm, thylakoid lumen, and P-OM in cyanobacteria.

Supplemental discussion:

Comparison of the thylakoid lumen proteome to previous thylakoid lumen proteomes

The thylakoid lumen proteome from this study was compared to the previously published thylakoid lumen proteome from PCC 7002 identified using APEX2 and a similar analysis method (Supplemental Fig. S5A) (Dahlgren et al., 2021). While the proteomes have an overlap of 70 proteins, differences in the identities of proteins detected by mass spectrometry and differences in the control proteins with a known thylakoid lumen localization explain the majority of the differences between the proteomes. Of the 35 thylakoid lumen proteins uniquely identified in this study, 15 were not identified by mass spectrometry in the previous study. Of the 53 proteins uniquely identified in the thylakoid lumen proteome of Dahlgren et al., 17 were not identified by mass spectrometry in this study (Dahlgren et al., 2021). Furthermore, thylakoid membrane proteins with multiple predicted transmembrane helices were excluded from the the list of known thylakoid lumen proteins in this study, but included as thylakoid lumen control proteins in the previous Dahlgren et al. study, so an additional 15 proteins with at least one predicted transmembrane helix that are likely localized to the thylakoid membrane were included in the thylakoid lumen proteome of Dahlgren et al. (Dahlgren et al., 2021). Furthermore, another 15 proteins uniquely identified in the thylakoid lumen by Dahlgren et al. were localized to the periplasm and not the thylakoid lumen in this study (Dahlgren et al., 2021). The difference in control proteins with a known thylakoid lumen localization used for analysis in each study is likely responsible for the number of proteins identified in the thylakoid lumen in the previous study by Dahlgren et al. that were identified as periplasmic in this study (Dahlgren et al., 2021). The thylakoid lumen proteome from this study was also compared to the recently published PSII-associated thylakoid lumen proteome of PCC 6803, which utilized a C-terminal APEX2 tag on PsbO (Xiao et al., 2022) (Supplemental Fig. S5B). While the analysis method for this study relied on control proteins with known localizations to identify enrichment cutoffs, Xiao et al. utilized enrichment cutoffs of a fold-change of 2 and statistical significance of $p<0.05$ on a student's t-test. When best reciprocal blast homologs are used to determine if each thylakoid lumen protein in PCC 7002 and PCC 6803 had an equivalent protein in other species, the overlap between the thylakoid lumen proteomes of this study (n = 105) and Xiao et al. (n = 131) was 45 proteins. While this overlap may seem small in comparison to the number of proteins identified in each study, only 80 of the thylakoid lumen proteins in this study had a homolog in PCC 6803 and only 86 of the thylakoid lumen proteins in Xiao et al. had a homolog in PCC 7002. Therefore, the fact that the studies were done in different species explains a large part of the differences between the thylakoid lumen proteomes. Xiao et al. identified 41 proteins in the thylakoid lumen with homologs in both species that were not in the thylakoid lumen proteome of this study. This study identified 35 proteins with homologs in both species in the thylakoid lumen that were not identified in the thylakoid lumen by Xiao et al. Differences in the identity of proteins detected by mass spectrometry explains a large portion of proteins with homologs in both species that were present in only one thylakoid lumen proteome. 14 out of the 35 unique

thylakoid lumen proteins with homologs identified in this study were not identified by mass spectrometry in any sample for Xiao et al. (Xiao et al., 2022). For Xiao et al., 17 out of 41 unique thylakoid lumen proteins with homologs were not identified in any of the mass spectrometry samples in this study (Xiao et al., 2022). For the remaining 24 proteins identified in the thylakoid lumen by Xiao et al., eight were localized to the periplasm only in this study (Dahlgren et al., 2021).

Supplemental Methods:

Membrane protein localization analysis

An analysis to identify membrane proteins using the cytoplasm as the FP list was not performed because membrane proteins can be labeled on both sides of the membrane. Exposure differences on each side of the membrane between proteins result in differential labeling on each side of the membrane, leading to high variation in enrichment values centered around an enrichment value of 0, making it difficult to obtain a high TPR-FPR value and draw a cutoff that is able to differentiate between membrane proteins with a specific localization and non-membrane proteins. Instead, to identify proteins that are preferentially localized to either the thylakoid lumen and membrane or the periplasm and outer or inner cell membranes, an analysis was performed comparing each thylakoid lumen replicate (PsbQ-APEX2 and A2695-APEX2) to each periplasm replicate (A1097-APEX2 and A1761-APEX2) for a total of 36 comparisons (Analysis 4). This avoided the issue of differential labeling on different sides of the membrane and allowed for high TPR-FPR values. The TP proteins included the TP list used for the thylakoid lumen in the thylakoid lumen vs cytoplasm analysis in addition to PCC 7002 proteins possessing at least one predicted transmembrane helix homologous to PCC 6803 proteins possessing at least one predicted transmembrane helix localized to the thylakoid membrane (Zak et al., 1999; Wang et al., 2000; Zak et al., 2001; Fulda et al., 2002; Ohkawa et al., 2002; Herranen et al., 2004; Zhang et al., 2004; Srivastava et al., 2005; Komenda et al., 2006; Pisareva et al., 2007; Xu et al., 2008; Boehm et al., 2009; Agarwal et al., 2010; Rowland et al., 2010; Pisareva et al., 2011; Rengstl et al., 2011; Roberts et al., 2012; Sacharz et al., 2015; Liberton et al., 2016; Selão et al., 2016; Zhu et al., 2016; Baers et al., 2019; Wang et al., 2022) in at least two studies that were identified in fewer inner membrane studies (Zak et al., 1999; Zak et al., 2001; Huang et al., 2002; Zhang et al., 2004; Huang et al., 2006; Pisareva et al., 2007; Rajalahti et al., 2007; Xu et al., 2008; Boehm et al., 2009; Schultze et al., 2009; Zhang et al., 2009; Pisareva et al., 2011; Wegener et al., 2011; Li et al., 2012; Roberts et al., 2012; Liberton et al., 2016; Selão et al., 2016; Zhu et al., 2016; Baers et al., 2019; Wang et al., 2022) than thylakoid membrane (Zak et al., 1999; Wang et al., 2000; Zak et al., 2001; Fulda et al., 2002; Ohkawa et al., 2002; Herranen et al., 2004; Zhang et al., 2004; Srivastava et al., 2005; Komenda et al., 2006; Pisareva et al., 2007; Xu et al., 2008; Boehm et al., 2009; Agarwal et al., 2010; Rowland et al., 2010; Pisareva et al., 2011; Rengstl et al., 2011; Roberts et al., 2012; Sacharz et al., 2015; Liberton et al., 2016; Selão et al., 2016; Zhu et al., 2016; Baers et al., 2019; Wang et al., 2022) and thylakoid lumen (Kashino et al., 2002; Kashino et al., 2006; Rajalahti et al., 2007; Aldridge et al., 2008; Schultze et al., 2009; Wegener et al., 2011; Heinz et al., 2016; Xiao et al., 2022) studies combined. Proteins in the TP list for the periplasm and outer membrane were excluded from the thylakoid membrane TP list. The FP list included the TP list used for the periplasm in the periplasm vs cytoplasm analysis in addition to PCC 7002 proteins possessing at least one predicted transmembrane helix homologous to PCC 6803 proteins localized to the plasma membrane (Zak et al., 1999; Zak et al., 2001; Huang et al., 2002; Zhang et al., 2004; Huang et al., 2006; Pisareva et al., 2007; Rajalahti et al., 2007; Xu et

al., 2008; Boehm et al., 2009; Schultze et al., 2009; Zhang et al., 2009; Pisareva et al., 2011; Wegener et al., 2011; Li et al., 2012; Roberts et al., 2012; Liberton et al., 2016; Selão et al., 2016; Zhu et al., 2016; Baers et al., 2019; Wang et al., 2022) in at least two studies and localized to the plasma membrane (Zak et al., 1999; Zak et al., 2001; Huang et al., 2002; Zhang et al., 2004; Huang et al., 2006; Pisareva et al., 2007; Rajalahti et al., 2007; Xu et al., 2008; Boehm et al., 2009; Schultze et al., 2009; Zhang et al., 2009; Pisareva et al., 2011; Wegener et al., 2011; Li et al., 2012; Roberts et al., 2012; Liberton et al., 2016; Selão et al., 2016; Zhu et al., 2016; Baers et al., 2019; Wang et al., 2022) more often than the thylakoid membrane. (Zak et al., 1999; Wang et al., 2000; Zak et al., 2001; Fulda et al., 2002; Ohkawa et al., 2002; Herranen et al., 2004; Zhang et al., 2004; Srivastava et al., 2005; Komenda et al., 2006; Pisareva et al., 2007; Xu et al., 2008; Boehm et al., 2009; Agarwal et al., 2010; Rowland et al., 2010; Pisareva et al., 2011; Rengstl et al., 2011; Roberts et al., 2012; Sacharz et al., 2015; Liberton et al., 2016; Selão et al., 2016; Zhu et al., 2016; Baers et al., 2019; Wang et al., 2022) Protein in the TP list for the thylakoid lumen were excluded from the FP list. The proteins included on this list are in Supplemental Table S2. Again, the TPR and FPR were calculated for each protein, and a cutoff was selected to maximize the difference between the TPR and FPR values. For a protein to be defined as localized to the thylakoid membrane or thylakoid lumen in this dataset, the protein must be above the cutoff in 35 or more of the 36 comparisons. A reverse analysis was performed to determine proteins localized to the periplasm, outer membrane, and inner membrane. Proteins above the cutoff in 35 or more of the 36 comparisons were defined as inner membrane, periplasm, or outer membrane in this analysis. Proteins that did not meet the cutoff for localization in either of these analyses were not able to be localized and categorized as unknown. Lastly, the same analysis described for the thylakoid membranes and inner membranes was performed using only the 261 proteins identified in this study with at least one transmembrane helix (Analysis 5).

Principal Component Analysis (PCA), K-means clustering, and data classification with machine learning

The PCA plots were made using log transformed normalized mass spectrometry values for each replicate of each sample in MATLAB. Unbiased k-means clustering was done in MATLAB using the mean enrichment values between each pair of APEX2 constructs as an input. The results of this analysis are in Supplemental Table S3. A support vector machine learning model in the scikit-learn v1.1.1 package in python v3.10 was trained using the mean enrichment values between each pair of APEX2 constructs as input values and the training classifications were control proteins from the thylakoid lumen, periplasm and OM, and cytoplasm. Any proteins in both the thylakoid lumen and periplasm and OM control list were excluded from the training data. After training, the support vector machine learning model was used to localize proteins to the cytoplasm, lumen, or periplasm and OM. Any protein with a probability of classification in the cytoplasm, lumen, or periplasm of less than 90% was classified as unknown. The machine learning classifications for each protein are listed in Supplemental Table S3.

Supplemental References:

Agarwal R, Matros A, Melzer M, Mock H-P, Sainis JK (2010) Heterogeneity in thylakoid membrane proteome of Synechocystis 6803. J Proteom 73: 976–991

Aldridge C, Spence E, Kirkilionis MA, Frigerio L, Robinson C (2008) Tat-dependent targeting of Rieske iron-sulphur proteins to both the plasma and thylakoid membranes in the cyanobacterium Synechocystis PCC6803. Mol Microbiol 70: 140–150

Baers LL, Breckels LM, Mills LA, Gatto L, Deery MJ, Stevens TJ, Howe CJ, Lilley KS, Lea-Smith DJ (2019) Proteome Mapping of a Cyanobacterium Reveals Distinct Compartment Organization and Cell-Dispersed Metabolism. Plant Physiol 181: 1721–1738

Belkin S, Mehlhorn RJ, Packer L (1987) Proton Gradients in Intact Cyanobacteria 1. Plant Physiol 84: 25–30

Boehm M, Nield J, Zhang P, Aro E-M, Komenda J, Nixon PJ (2009) Structural and Mutational Analysis of Band 7 Proteins in the Cyanobacterium Synechocystis sp. Strain PCC 6803. J Bacteriol 191: 6425–6435

Cameron JC, Wilson SC, Bernstein SL, Kerfeld CA (2013) Biogenesis of a Bacterial Organelle: The Carboxysome Assembly Pathway. Cell 155: 1131–1140

Dahlgren KK, Gates C, Lee T, Cameron JC (2021) Proximity-based proteomics reveals the thylakoid lumen proteome in the cyanobacterium Synechococcus sp. PCC 7002. Photosynth Res 147: 177–195

Dinh T, Bernhardt TG (2011) Using Superfolder Green Fluorescent Protein for Periplasmic Protein Localization Studies. J Bacteriol 193: 4984–4987

Dong L-L, Li Q-D, Wu D, Sun Y-F, Zhou M, Zhao K-H (2016) A novel periplasmic protein (Slr0280) tunes photomixotrophic growth of the cyanobacterium, Synechocystis sp. PCC 6803. Gene 575: 313–320

Fulda S, Huang F, Nilsson F, Hagemann M, Norling B (2000) Proteomics of Synechocystis sp. strain PCC 6803. Eur J Biochem 267: 5900–5907

Fulda S, Norling B, Schoor A, Hagemann M (2002) The Slr0924 protein of Synechocystis sp. strain PCC 6803 resembles a subunit of the chloroplast protein import complex and is mainly localized in the thylakoid lumen. Plant Mol Biol 49: 107–118

Heinz S, Liauw P, Nickelsen J, Nowaczyk M (2016) Analysis of photosystem II biogenesis in cyanobacteria. Biochim Biophys Acta Bioenerg 1857: 274–287

Herranen M, Battchikova N, Zhang P, Graf A, Sirpiö S, Paakkarinen V, Aro E-M (2004) Towards Functional Proteomics of Membrane Protein Complexes in Synechocystis sp. PCC 6803. Plant Physiol 134: 470–481

Huang F, Fulda S, Hagemann M, Norling B (2006) Proteomic screening of salt-stress-induced changes in plasma membranes of Synechocystis sp. strain PCC 6803. Proteomics 6: 910–920

Huang F, Parmryd I, Nilsson F, Persson AL, Pakrasi HB, Andersson B, Norling B (2002) Proteomics of Synechocystis sp. Strain PCC 6803: Identification of Plasma Membrane Proteins. Mol Cell Proteomics 1: 956–966

Kashino Y, Inoue-Kashino N, Roose JL, Pakrasi HB (2006) Absence of the PsbQ Protein Results in Destabilization of the PsbV Protein and Decreased Oxygen Evolution Activity in Cyanobacterial Photosystem II. J Biol Chem 281: 20834–20841

Kashino Y, Lauber WM, Carroll JA, Wang Q, Whitmarsh J, Satoh K, Pakrasi HB (2002) Proteomic Analysis of a Highly Active Photosystem II Preparation from the Cyanobacterium Synechocystis sp. PCC 6803 Reveals the Presence of Novel Polypeptides. Biochemistry 41: 8004–8012

Klinkert B, Ossenbühl F, Sikorski M, Berry S, Eichacker L, Nickelsen J (2004) PratA, a Periplasmic Tetratricopeptide Repeat Protein Involved in Biogenesis of Photosystem II in Synechocystis sp. PCC 6803. J Biol Chem 279: 44639–44644

Komenda J, Barker M, Kuviková S, Vries R de, Mullineaux CW, Tichý M, Nixon PJ (2006) The FtsH Protease slr0228 Is Important for Quality Control of Photosystem II in the Thylakoid Membrane of Synechocystis sp. PCC 6803. J Biol Chem 281: 1145–1151

Kurian D, Phadwal K, Mäenpää P (2006) Proteomic characterization of acid stress response in Synechocystis sp. PCC 6803. Proteomics 6: 3614–3624

Li T, Yang H-M, Cui S-X, Suzuki I, Zhang L-F, Li L, Bo T-T, Wang J, Murata N, Huang F (2012) Proteomic Study of the Impact of Hik33 Mutation in Synechocystis sp. PCC 6803 under Normal and Salt Stress Conditions. J Proteome Res 11: 502–514

Liberton M, Page LE, O'Dell WB, O'Neill H, Mamontov E, Urban VS, Pakrasi HB (2013) Organization and Flexibility of Cyanobacterial Thylakoid Membranes Examined by Neutron Scattering. J Biol Chem 288: 3632–3640

Liberton M, Saha R, Jacobs JM, Nguyen AY, Gritsenko MA, Smith RD, Koppenaal DW, Pakrasi HB (2016) Global Proteomic Analysis Reveals an Exclusive Role of Thylakoid Membranes in Bioenergetics of a Model Cyanobacterium. Mol Cell Proteomics 15: 2021–2032

Ohkawa H, Sonoda M, Hagino N, Shibata M, Pakrasi HB, Ogawa T (2002) Functionally distinct NAD (P)H dehydrogenases and their membrane localization in Synechocystis sp. PCC6803. Functional Plant Biol 29: 195–200

Pisareva T, Kwon J, Oh J, Kim S, Ge C, Wieslander Å, Choi J-S, Norling B (2011) Model for Membrane Organization and Protein Sorting in the Cyanobacterium Synechocystis sp. PCC 6803 Inferred from Proteomics and Multivariate Sequence Analyses. J Proteome Res 10: 3617–3631

Pisareva T, Shumskaya M, Maddalo G, Ilag L, Norling B (2007) Proteomics of Synechocystis sp. PCC 6803. FEBS J 274: 791–804

Rajalahti T, Huang F, Rosén Klement M, Pisareva T, Edman M, Sjöström M, Wieslander Å, Norling B (2007) Proteins in Different Synechocystis Compartments Have Distinguishing N-Terminal Features: A Combined Proteomics and Multivariate Sequence Analysis. J Proteome Res 6: 2420–2434

Rengstl B, Oster U, Stengel A, Nickelsen J (2011) An Intermediate Membrane Subfraction in Cyanobacteria Is Involved in an Assembly Network for Photosystem II Biogenesis. J Biol Chem 286: 21944–21951

Rhee H-W, Zou P, Udeshi ND, Martell JD, Mootha VK, Carr SA, Ting AY (2013) Proteomic Mapping of Mitochondria in Living Cells via Spatially Restricted Enzymatic Tagging. Science 339: 1328–1331

Roberts IN, Lam XT, Miranda H, Kieselbach T, Funk C (2012) Degradation of PsbO by the Deg Protease HhoA Is Thioredoxin Dependent. PLOS One 7: e45713

Rowland JG, Simon WJ, Nishiyama Y, Slabas AR (2010) Differential proteomic analysis using iTRAQ reveals changes in thylakoids associated with Photosystem II-acquired thermotolerance in Synechocystis sp. PCC 6803. Proteomics 10: 1917–1929

Sacharz J, Bryan SJ, Yu J, Burroughs NJ, Spence EM, Nixon PJ, Mullineaux CW (2015) Sub-cellular location of FtsH proteases in the cyanobacterium Synechocystis sp. PCC 6803 suggests localised PSII repair zones in the thylakoid membranes. Mol Microbiol 96: 448–462

Schultze M, Forberich B, Rexroth S, Dyczmons NG, Roegner M, Appel J (2009) Localization of cytochrome b6f complexes implies an incomplete respiratory chain in cytoplasmic membranes of the cyanobacterium Synechocystis sp. PCC 6803. Biochim Biophys Acta Bioenerg 1787: 1479–1485

Selão TT, Zhang L, Knoppová J, Komenda J, Norling B (2016) Photosystem II Assembly Steps Take Place in the Thylakoid Membrane of the Cyanobacterium Synechocystis sp. PCC6803. Plant Cell Physiol 57: 95–104

Srivastava R, Pisareva T, Norling B (2005) Proteomic studies of the thylakoid membrane of Synechocystis sp. PCC 6803. Proteomics 5: 4905–4916

Stoddard A, Rolland V (2019) I see the light! Fluorescent proteins suitable for cell wall/apoplast targeting in Nicotiana benthamiana leaves. Plant Direct 3: e00112

Veit S, Takeda K, Tsunoyama Y, Baymann F, Nevo R, Reich Z, Rögner M, Miki K, Rexroth S (2016) Structural and functional characterisation of the cyanobacterial PetC3 Rieske protein family. Biochim Biophys Acta Bioenerg 1857: 1879–1891

Wang J, Huang X, Ge H, Wang Y, Chen W, Zheng L, Huang C, Yang H, Li L, Sui N, et al (2022) The quantitative proteome atlas of a model cyanobacterium. J Genet Genom 49: 96–108

Wang Y, Sun J, Chitnis PR (2000) Proteomic study of the peripheral proteins from thylakoid membranes of the cyanobacterium Synechocystis sp. PCC 6803. Electrophoresis 21: 1746–1754

Wegener KM, Bennewitz S, Oelmüller R, Pakrasi HB (2011) The Psb32 Protein Aids in Repairing Photodamaged Photosystem II in the Cyanobacterium Synechocystis 6803. Mol Plant 4: 1052–1061

Xiao Z, Huang C, Ge H, Wang Y, Duan X, Wang G, Zheng L, Dong J, Huang X, Zhang Y, et al (2022) Proximity Labeling Facilitates Defining the Proteome Neighborhood of Photosystem II Oxygen Evolution Complex in a Model Cyanobacterium. Mol Cell Proteomics 21: 100440

Xu M, Ogawa T, Pakrasi HB, Mi H (2008) Identification and Localization of the CupB Protein Involved in Constitutive CO2 Uptake in the Cyanobacterium, Synechocystis sp. Strain PCC 6803. Plant Cell Physiol 49: 994–997

Zak E, Norling B, Andersson B, Pakrasi HB (1999) Subcellular localization of the BtpA protein in the cyanobacterium Synechocystis sp. PCC 6803. Eur J Biochem 261: 311–316

Zak E, Norling B, Maitra R, Huang F, Andersson B, Pakrasi HB (2001) The initial steps of biogenesis of cyanobacterial photosystems occur in plasma membranes. PNAS 98: 13443–13448

Zhang L-F, Yang H-M, Cui S-X, Hu J, Wang J, Kuang T-Y, Norling B, Huang F (2009) Proteomic Analysis of Plasma Membranes of Cyanobacterium Synechocystis sp. Strain PCC 6803 in Response to High pH Stress. J Proteome Res 8: 2892–2902

Zhang P, Battchikova N, Jansen T, Appel J, Ogawa T, Aro E-M (2004) Expression and Functional Roles of the Two Distinct NDH-1 Complexes and the Carbon Acquisition Complex NdhD3/NdhF3/CupA/Sll1735 in Synechocystis sp PCC 6803. Plant Cell 16: 3326–3340

Zhu Y, Liberton M, Pakrasi HB (2016) A Novel Redoxin in the Thylakoid Membrane Regulates the Titer of Photosystem I. J Biol Chem 291: 18689–18699
