# Supplementary figures and images for: Spatial proteomics reveals signal sequence characteristics correlated with localization in cyanobacteria
Source: Plant Physiol. 2025 Aug 6;198(4):kiaf186. doi: 10.1093/plphys/kiaf186 (PMC12341915; doi:10.1093/plphys/kiaf186)

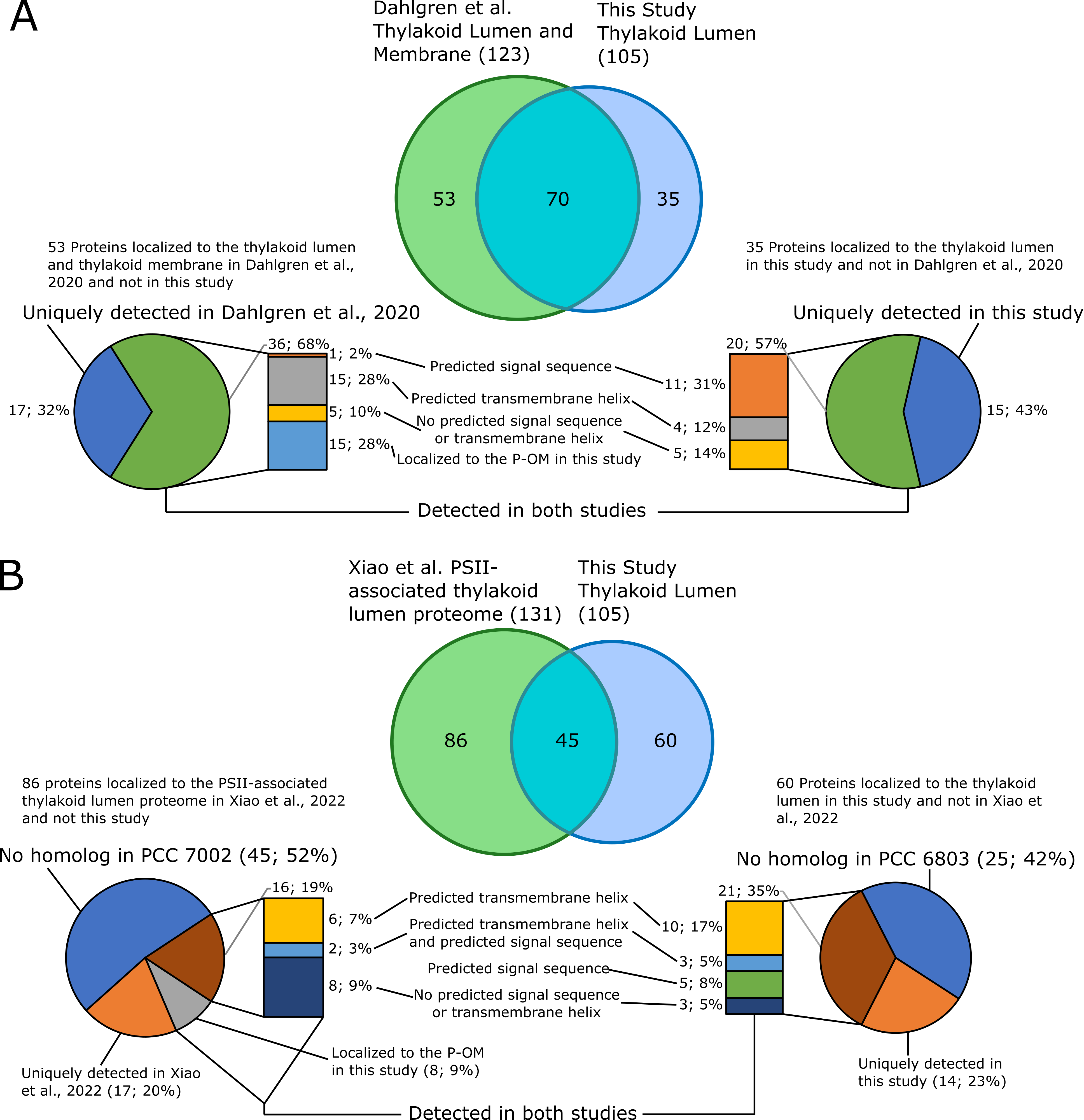

Supplement: kiaf186_Supplementary_Data [file kiaf186_supplementary_data.zip › Fig S5 - Comparison between lumen proteomes 600 dpi.tiff]

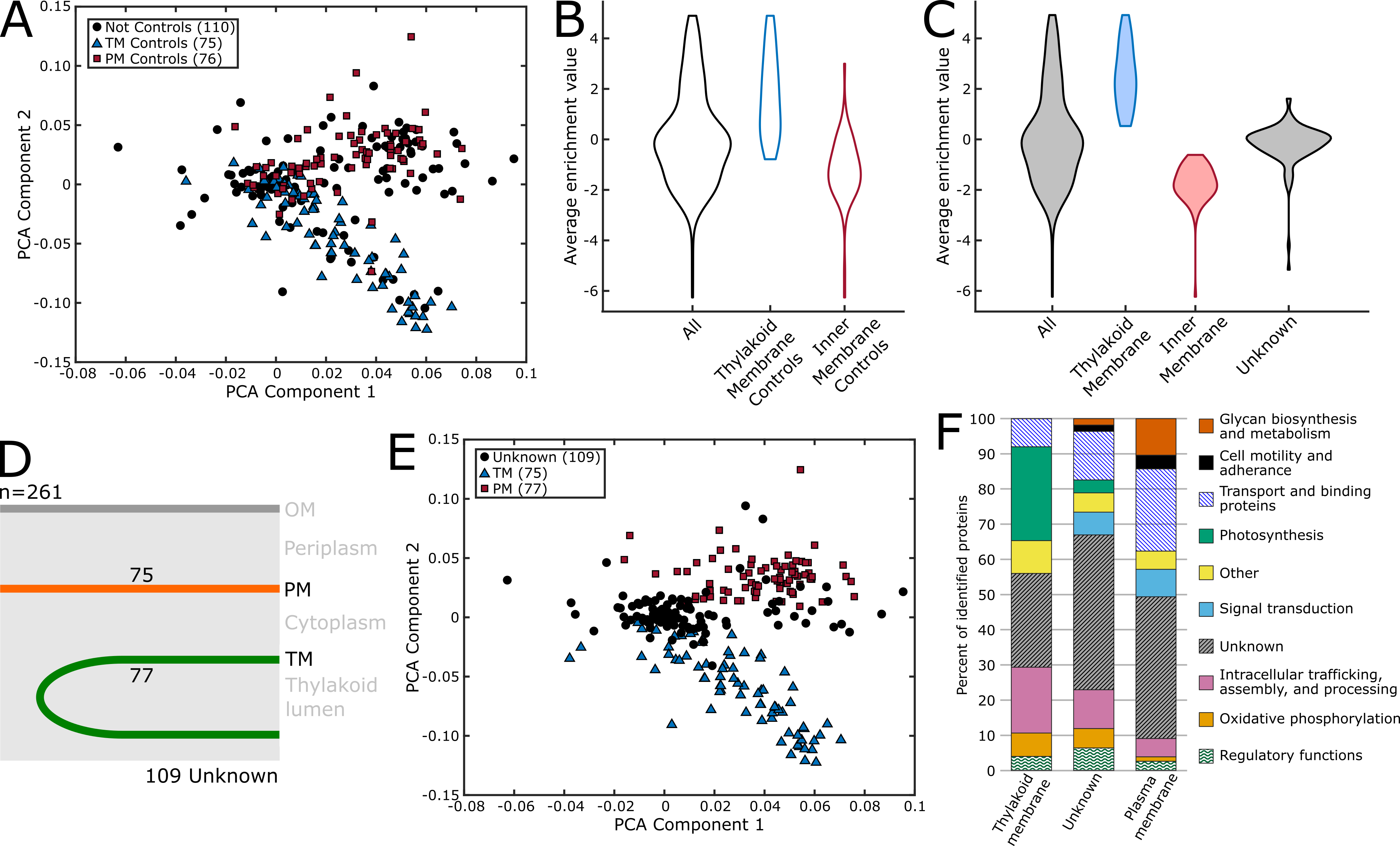

Supplement: kiaf186_Supplementary_Data [file kiaf186_supplementary_data.zip › Fig S8 - membrane proteomics analysis 600 dpi.tiff]
